# Excited State Anions in Organic Transformations

**DOI:** 10.1002/anie.202009288

**Published:** 2020-12-29

**Authors:** Matthias Schmalzbauer, Michela Marcon, Burkhard König

**Affiliations:** ^1^ Faculty of Chemistry and Pharmacy University of Regensburg Universitätsstrasse 31 93053 Regensburg Germany

**Keywords:** excited anions, electron transfer, photoredox catalysis, photoreduction, synthetic photochemistry

## Abstract

Utilizing light is a smart way to fuel chemical transformations as it allows the energy to be selectively focused on certain molecules. Many reactions involving electronically excited species proceed via open‐shell intermediates, which offer novel and unique routes to expand the hitherto used synthetic toolbox in organic chemistry. The direct conversion of non‐prefunctionalized, less activated compounds is a highly desirable goal to pave the way towards more sustainable and atom‐economic chemical processes. Photoexcited closed‐shell anions have been shown to reach extreme potentials in single electron transfer reactions and reveal unusual excited‐state reactivity. It is, therefore, surprising that their use as a reagent or photocatalyst is limited to a few examples. In this Review, we briefly discuss the characteristics of anionic photochemistry, highlight pioneering work, and show recent progress which has been made by utilizing photoexcited anionic species in organic synthesis.

## Graphical abstract

Photoactivated anions act as powerful single‐electron reductants and can be used as either a catalyst or a reactant. In the presence of a base, their absorption is shifted bathochromically compared to the neutral molecules through deprotonation, with visible light often being sufficient for their excitation.

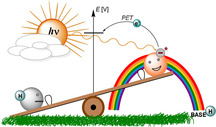

## 1 Introduction

Initial attention to the versatile reaction modes of photoexcited organic anions and their special spectroscopic behavior was drawn by the early reviews of Fox^[1]^ and Tolbert.^[2]^ Since then, other excellent publications followed that summarized the photochemistry of excited organic anions with a focus on their photoreductive properties and underlining the peculiarities of anionic organic molecules in photochemistry.[^3^, ^4^] Compared to the neutral species, the absorption of an organic anion is usually red‐shifted, which facilitates selective excitation in complex mixtures and often allows visible light to be used. Along with the enhanced electron–electron repulsion found in anionic molecules, negatively charged species are expected to act as particularly potent electron donors from their photoexcited states. In addition, a single‐electron transfer from an anionic donor to a neutral acceptor gives rise to a neutral radical and a radical anion. These species are free of attracting forces and are able to diffuse freely, which suppresses back electron transfer (BET) reactions and results in higher reaction efficiencies. Organic anions can be easily formed in the presence of base, and their rather long excited‐state lifetimes distinguish them from radical anions.

Excited anionic species are also utilized in key photochemical steps in biology. For example, in an ATP‐driven process, the excited oxyluciferin anion causes the bioluminescence of fireflies.^[5]^ Moreover, phototrophic organisms show locomotory movement upon stimulus by light. The photoactive yellow protein (PYP) encloses the anionic *trans*‐*para*‐coumaric acid as a blue‐light photoreceptor. Subsequent *trans*–*cis* isomerization of the excited chromophore induces a conformational change of the protein leading to a biological signal transduction.^[6]^ The enzyme‐mediated repair of photodamaged DNA is another well‐known example of excited anions in living cells. A crucial step is the photoinduced electron transfer from the excited cofactor flavin adenine dinucleotide (FADH^−^), which provides an electron for the light‐driven repair catalyzed by photolyases.[^7^, ^8^]

The last decade has been a very exciting time in terms of photochemistry, and many novel chemical transformations have been developed which complement the available synthetic repertoire. We are sure that, inspired by nature and the herein‐presented examples, the photochemistry of closed‐shell anions will be further developed towards the generation of ever stronger light‐activated reductants and novel reaction modes. In this Review, we briefly summarize key spectroscopic and electrochemical properties of organic anions and provide an overview of the versatile photochemistry of anionic species with a special focus on recent examples of the use of organic anions as photocatalysts or as light‐activated reagents.

### 1.1 Spectroscopic Properties of Organic Anions

**Figure 1:**
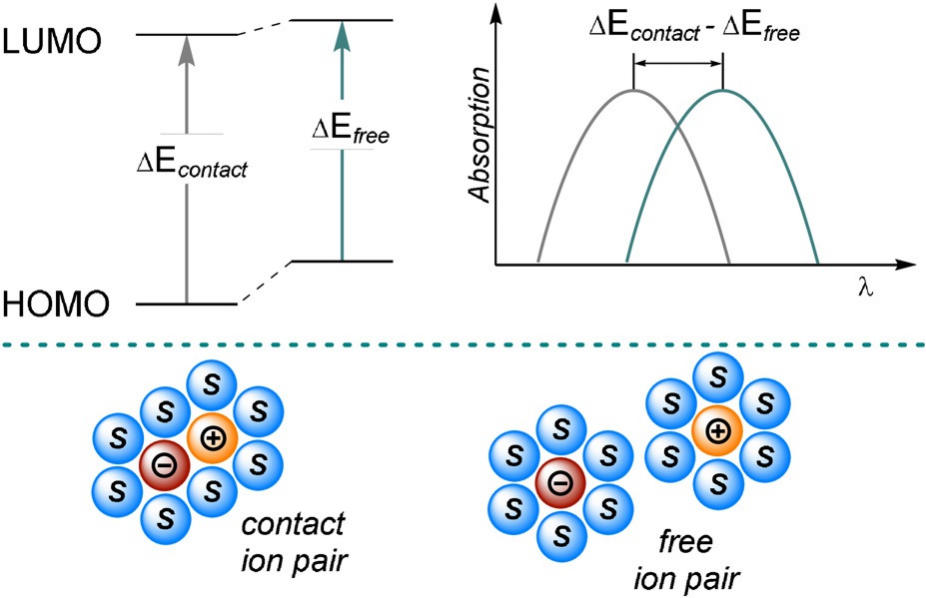
Energetic destabilization of the ground state of a free ion pair in polar solvent compared to the contact ion pair in a nonpolar solvent and influence on the absorption spectrum.

The chemistry of molecules excited by light is initiated by the absorption of a photon and, thus, we will start by discussing the peculiarities of the absorption spectra of closed‐shell anions. Compared to their neutral precursors, organic anions usually experience a significant bathochromic shift of their absorption spectra, and pronounced absorption bands can be attributed to π,π* transitions. The narrowed gap between the highest occupied molecular orbital (HOMO) and the lowest unoccupied molecular orbital (LUMO, Figure 1) causing the red‐shift can be primarily explained by the increased shielding of the core because of an imbalance of charges. The strength of the electric field is reduced and electrons in the HOMO sense much weaker attracting forces. As a result, the spatial distribution of electrons becomes more diffuse as the conjugation length is extended.[^1^, ^9^] The absorption of organic anions is also affected by the size and nature of the countercation, solvent polarity, and ion‐pairing effects in solution. In nonpolar or weakly polar solvents, contact ion pairs are formed and the properties of the anionic species are strongly influenced by the character of the countercation.

In contrast, the increased solubility of ions in polar solvents, induced by aligning molecular dipoles, causes solvent‐separated or free ion pairs and the mutual ionic interaction is diminished. In general, an increase in the solvent polarity and/or the ionic radius of the countercation results in a bathochromic shift of the absorption, which can be attributed to a destabilization of the ground‐state ion pair. This destabilization effect is less pronounced in the excited state.[^1^, ^4^]

Similarly, the emission of excited organic anions is usually influenced by the solvent polarity and countercation. The fluorescence decay of sodium 2‐naphtholate was studied in different solvents, for example.^[10]^ For polar protic and polar aprotic solvents, a monoexponential fluorescence decay was observed. However, the fluorescence lifetime in polar protic MeOH was remarkably decreased and the emission spectrum was blue‐shifted compared to those in polar aprotic DMF or DMSO, which the authors attribute to a stabilization of the anion ground state caused by a strong hydrogen bonding of the solvent. In weakly polar THF, contact ion pairs and solvent‐separated ion pairs of 2‐naphtholate and Na^+^ coexist and cause a biexponential fluorescence decay because of their varying fluorescence lifetimes. The addition of crown ether to the system led to a monoexponential decay being recorded, which suggested that sodium cations are complexed and the ion pairs formed with naphtholate are solvent‐separated in nature. Owing to the lack of ground‐state stabilization in solvent‐separated or free ion pairs, lifetimes similar to those obtained from experiments in polar aprotic solvents were found in the presence of a crown ether.

**Scheme 1:**
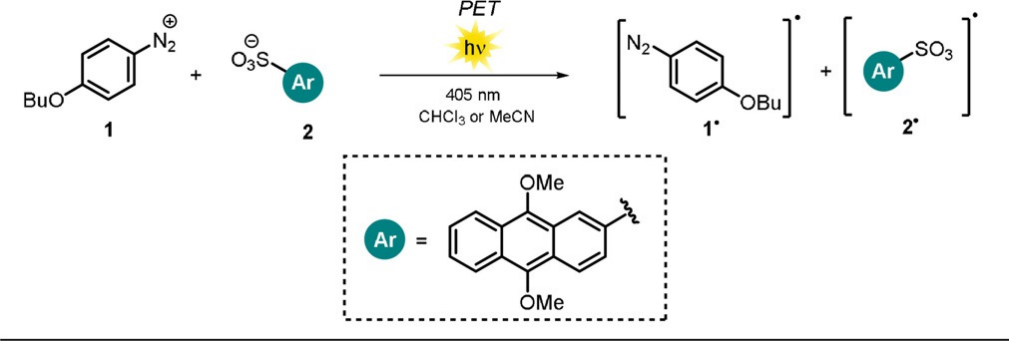
The rate of photoinduced electron transfer is influenced by the solvent polarity: fast in CHCl_3_ (tight pair), slow in MeCN (loose pair).

The nature of ion pairing might also affect the efficiency of bimolecular electron‐transfer processes. Tamaoki et al. studied the quantum yield for the photodissociation of the benzene diazonium salt **1** in the presence of 9,10‐dimethoxyanthracene‐2‐sulfonate (**2**) as the visible‐light‐absorbing counteranion (Scheme 1).^[11]^ The photodecomposition of the benzene diazonium cation **1** initiated by photoinduced electron transfer (PET) from the excited anion **2** was found to be six‐times higher in CHCl_3_ than in MeCN. The difference in the reactivity of the diazonium salt in the solvents was explained by the different nature of the ion pairs formed. The weakly polar solvent CHCl_3_ promotes a fast reaction because of the proximity of **1** and **2** in a tight ion pair. Solvent‐separated loose ion pairs in polar MeCN allowed a distinct fluorescence lifetime to be measured. Upon excitation in polar media, the anionic donor needs to initially encounter a cationic acceptor to trigger the photodecomposition and, hence, increased lifetimes are recorded. For a more comprehensive discussion of ion‐pairing and solvent effects, we refer to several excellent reports.[^4^, ^10^, ^12^, ^13^, ^14^]

### 1.2 Photoinduced Electron Transfer

**Figure 2:**
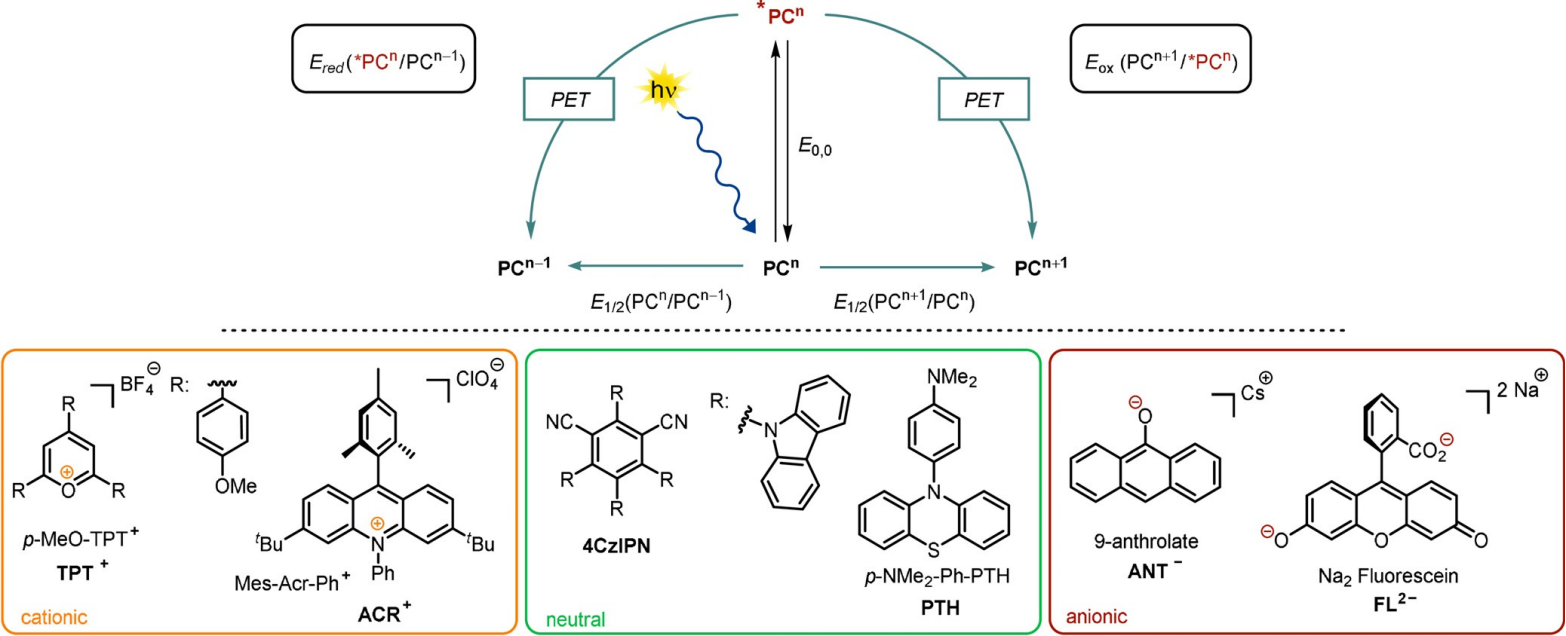
Diagram of the ground‐ and excited‐state potentials of a photocatalyst (PC, top). Representatives of cationic, neutral, and anionic organic photocatalysts (bottom).

Electron‐transfer reactions from electronically excited states of molecules were among the earliest photochemical reactions reported.^[15]^ Photoexcited molecules exhibit increased reduction and oxidation potentials compared to their ground states and the resulting excited‐state potentials can be estimated, according to the free enthalpy change of a PET, by measuring the ground‐state potentials *E*_1/2_ and the transition energy *E*_0,0_ (Figure 2).^[16]^ In polar organic solvents, the electrostatic work term usually contributes little to the free enthalpy change and is frequently omitted.^[17]^

**Scheme 2:**
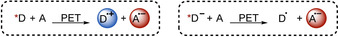
Charge separation with a neutral donor (left) and charge shift with an anionic donor (right).

PET from a neutral excited‐state donor (^*^D) to a neutral ground‐state acceptor (A) causes a charge separation, thereby resulting in a pair of radical ions. In contrast, PET from an anionic excited‐state donor to a neutral acceptor can be considered as a charge shift, generating products that are free of electrostatic attraction and expected to diffuse freely (Scheme 2). Hence, the lost channel of a back electron transfer, which would regenerate the initial non‐excited starting materials, is less competitive in a charge‐shift process.^[18]^

An anionic molecule is considered to be a more superior electron donor than its neutral parent compound as both the repulsion between electrons and the shielding from the nucleus are increased. As a consequence, the excess negative charge facilitates the removal of an electron. Experimentally, this becomes apparent when solvated electrons are expelled from organic anions in a biphotonic process using energy‐rich UV light^[19]^ in glassy matrices (77 K) or pulsed high‐energy lasers[^20^, ^21^] in alkaline aqueous solution. Working with visible‐light‐emitting diodes (LEDs) and common organic solvents, however, renders the photoejection of an electron unlikely to occur and, hence, electron‐transfer reactions prevail under these conditions. We recently demonstrated that 9‐anthrone and its derivatives are easily deprotonated in the presence of a carbonate base to form colored anions (e.g. **ANT^−^**, Figure 2), which upon excitation with visible light turn into remarkably strong reductants.^[22]^ Cyclic voltammetry measurements in alkaline DMSO revealed that the anionic ground state is already a good reductant, as the excess charge is removed easily because of resonance stabilization of the resulting radical. In sharp contrast, the dianions of fluorescein **FL^2−^** or eosin Y (**EY^2−^**) show a significantly decreased tendency towards electrochemical oxidation in alkaline MeOH and, hence, the resulting excited‐state oxidation potentials are only moderate (Table 1, entries 5–7).^[23]^

**Table 1:** Ground‐state (*E*_1/2_) and excited‐state (*E*_red_, *E*_ox_) redox potentials of selected cationic, neutral, and anionic photocatalysts (PC) and the corresponding transition energies (*E*_0,0_).

Entry	PC^*n*^	*E*_1/2_(*PC^n^/PC* ^*n*−1^) [eV]	*E*_red_(*PC^n^/PC ^*n*−1^) [eV]	*E*_1/2_(*PC* ^*n*+1^ */PC* ^*n*^) [eV]	*E*_ox_(*PC* ^*n*+1^ /*PC ^*n*^) [eV]	*E*_0,0_ [eV]
						
1^[17]^	**TPT^+^**	−0.50^[a]^	+1.84	–	–	2.34^[b]^
2^[30]^	**ACR^+^**	−0.59^[c]^	+2.08	–	–	2.67
						
3^[31]^	**4CzIPN**	−1.24^[d]^	+1.43	+1.49^[d]^	−1.18	2.67
4^[32]^	**PTH**	–	–	+0.57^[d]^	−2.5	3.1
						
5^[22]^	**ANT^−^**	–	–	−0.34^[d,e]^	−2.65	2.31
6^[23]^	**FL^2−^**	–	–	+0.87^[f]^	−1.55	2.42
7^[17]^	**EY^2−^**	−1.06	+1.23^[g,h]^	+0.76	−1.58^[g,h]^	2.31^[g]^
			+0.83^[i,h]^		−1.08^[i,h]^	1.91^[h]^
8^[72]^	**PhPH^−^**	–	–	−0.10^[e]^	−3.16	3.06
9^[79]^	**BIA‐H.1^−^**	–	–	+0.06^[j]^	−2.71	2.77^[k]^
10^[86]^	**TMA^−^**	–	–	−0.51^[d,e]^	−2.92	2.41

Potentials are reported vs. saturated calomel electrode (SCE). The transition energy *E*_0,0_ was determined from the intersection of the normalized absorption and emission spectra. [a] Potential recorded vs. normal hydrogen electrode (NHE) and converted into vs. the SCE by subtracting 0.141 V. [b] Determined from the lowest energy emission maximum. [c] Potential recorded vs. Ag/AgCl and converted into vs. SCE by subtracting 0.03 V. [d] Recorded vs. the ferrocene redox couple (Fc^+^/Fc) and converted into vs. SCE by adding 0.38 V. [e] Potential was measured in dry degassed DMSO with excess of Cs_2_CO_3_. [f] Measured in MeOH containing NaOH (0.1 mm) against Ag/AgCl and referenced to SCE by conversion. [g] Values for the singlet excited state. [h] Potential recorded vs. Ag/AgCl and converted to vs. SCE by subtracting 0.039 V. [i] Values for triplet excited state. [j] Potential measured in MeCN with excess ^*t*^BuOK. [k] Estimated by the end absorption wavelength with an absorbance of 0.02 at 4.0×10^−5^ m.

**Scheme 3:**
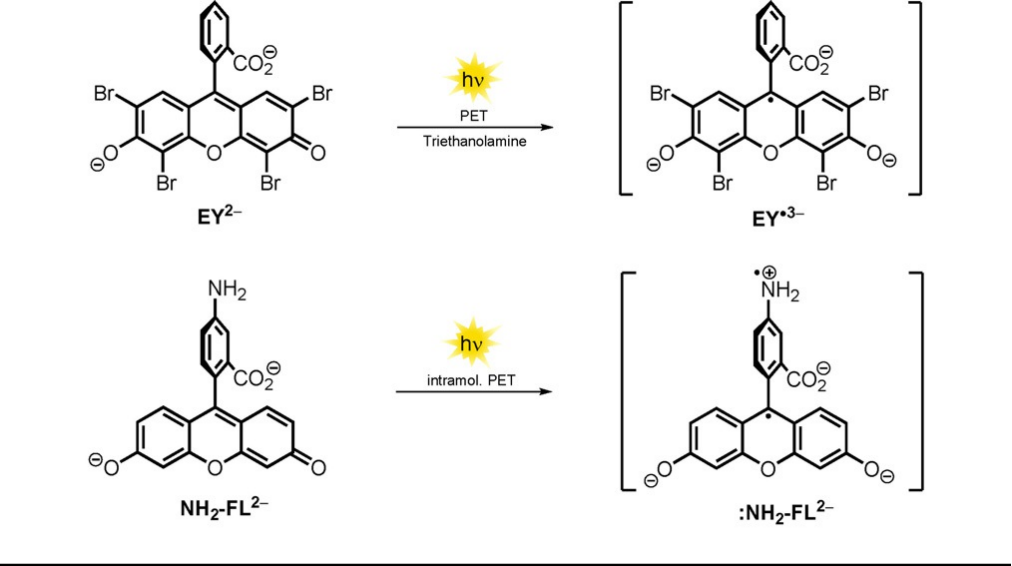
Formation of the eosin Y radical trianion upon PET in the presence of triethanolamine (top). Intramolecular PET from the amino group causing self‐quenching of the fluorescence (bottom).

Furthermore, it was reported that **FL^2−^** and **EY^2−^**, although being present as ground‐state dianions, are easily reduced upon photoexcitation in basic solutions containing triethanolamine or phenol to form radical trianions (Scheme 3, top).[^24^, ^25^, ^26^, ^27^, ^28^] Walt and co‐workers attached an amino group to the benzoate scaffold of fluorescein **NH_2_‐FL^2−^** and found that the fluorescence quantum yield dropped by almost a factor of 60. They explained this observation by an intramolecular PET from the nitrogen lone pair of electrons to the fluorescein scaffold (Scheme 3, bottom). A similar fluorescence quantum yield with respect to unmodified **FL^2−^** was, however, recorded when adjusting the pH value of the solution to around the p*K_a_* value of the aromatic amine. As a consequence of protonation of the amine, the nitrogen lone pair is no longer available for intramolecular PET, thereby resulting in increased fluorescence.^[29]^ In 1991, Soumillion and co‐workers showed that the fluorescence of the excited anion of the xanthene dye resorufin is quenched in the presence of 2‐naphtholate, and the formation of a radical dianion of resorufin was proposed.^[18]^ The moderate reducing abilities of negatively charged xanthene dyes (e.g. **EY^2−^**, **FL^2−^**) can be explained by an overwhelming contribution of the electron‐deficient conjugated system to the overall electronic properties. Thus, to obtain strongly reducing excited anions, a facile single‐electron oxidation is crucial (Table 1, entries 5 and 6 show similar values for *E*_0,0_, but differ significantly in their ground‐state and excited‐state oxidation potentials).

## 2 Anionic Compounds as Photocatalysts

### 2.1 Photoredox Catalysis

During the last decade, impressive progress has been made in the field of synthetic photoredox catalysis, and many novel transformations which were previously inaccessible have been developed. Photoexciting a molecule changes the electron distribution in the molecular orbitals, thereby resulting in the excited species having both increased oxidizing and reducing abilities compared to the ground state (see Figure 2). These redox properties can be fine‐tuned by attaching electron‐donating or ‐withdrawing substituents.[^30^, ^31^, ^32^, ^33^] Up to now, a variety of photocatalysts have been reported and these are often classified in terms of their composition as polypyridyl transition metal complexes,^[34]^ organic dyes,^[17]^ or polyoxometalates^[35]^ (POMs). In addition, heterogeneous organic semiconductors have been successfully employed as photocatalysts.^[36]^ Their intrinsic photophysical properties such as the redox potential of the excited state, absorption of light, and the lifetime of the excited state define the scope and limitations in chemical reactions. Selected examples of organic photocatalysts are depicted in Figure 2. The photochemistry of the uncharged donor–acceptor dyad **4CzIPN** covers a broad electrochemical range (see Table 1, entry 3). As a result of the versatile chemistry arising from its excited state, it is often used to replace precious and toxic Ru‐ or Ir‐polypyridyl complexes.[^31^, ^37^] However, higher excited‐state potentials need to be achieved to convert less activated substrates through photoinduced single‐electron transfer. Recently, it was shown that photoexcited, electron‐rich *N*‐arylphenothiazines (e.g. **PTH**) act as very strong reductants, but these compounds do not absorb in the visible range and hence UV light is necessary, which might interfere with other reaction components. Large Stokes shifts were found for the substituted *N*‐arylphenothiazines, which result in high values for the transition energy (see Table 1, entry 4).^[32]^

**Scheme 4:**
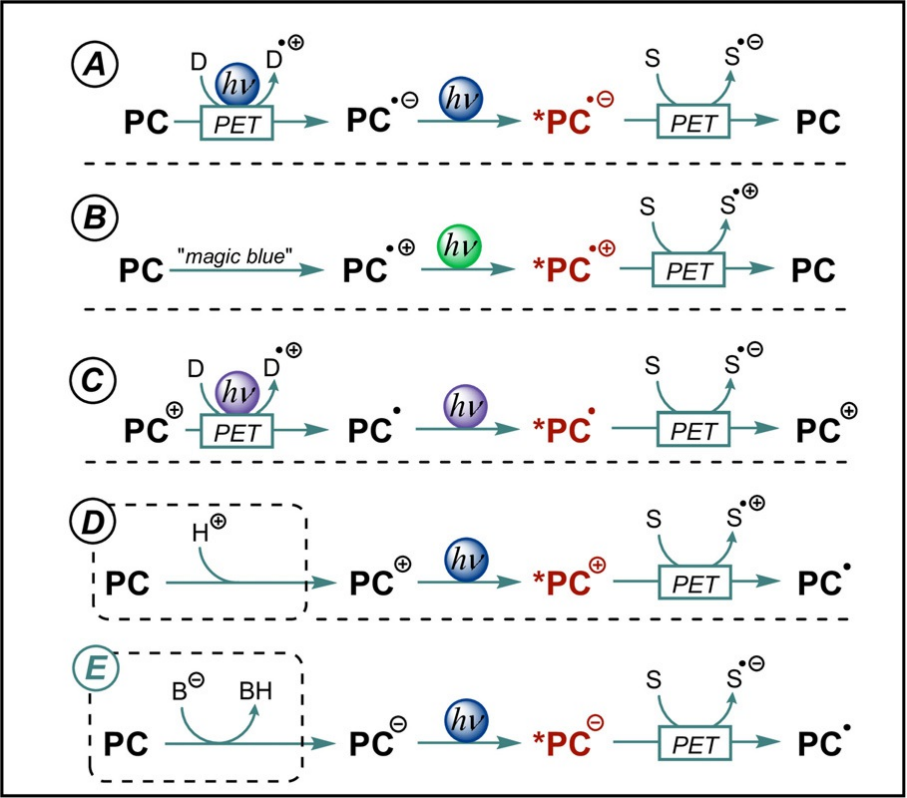
Approaches leading to reactive excited‐state photocatalysts with extreme redox potentials (red) that allow non‐activated substrates (S) to be converted. The initial activation by protonation/deprotonation (see D, E) is not required when the salt of the catalyst is directly used.

Apart from commonly used neutral organic dyes, molecules with a charged or an open‐shell ground state or both were found to significantly increase achievable excited‐state potentials and allowed the substrate scope to be widened for photoinduced electron‐transfer reactions (Scheme 4). Several organic dyes form stable and colored radical anions through PET in the presence of suitable sacrificial donors and, hence, enable a subsequent second excitation (see Scheme 4 A).[^38^, ^39^, ^40^, ^41^] The versatile photochemistry of excited radical anions allowed various (hetero)aryl halides to be converted in coupling reactions and has been the subject of several reviews.[^42^, ^43^, ^44^, ^45^] Very recently, this strategy was reported to promote Birch‐type reductions of benzene derivatives upon irradiation with visible light.^[46]^ In contrast, the formation of super‐oxidants has been reported upon photoexcitation of stable, chemically generated phenothiazine radical cations (Scheme 4 B).^[47]^ Furthermore, electron transfer from the photoexcited doublet states of neutral radicals has been studied.[^48^, ^49^, ^50^, ^51^, ^52^] The acridine radical **ACR^.^** was recently found to act as an extremely potent photoreductant upon excitation with black light (Scheme 4 C).^[53]^ Although excited open‐shell species offer high redox potentials, their photochemistry suffers from short lifetimes, which are usually in the picosecond range.[^53^, ^54^, ^55^] As the photochemistry of open‐shell molecules is beyond the scope of this Review, the interested reader is referred to cited literature.

Photoreactions using catalytic amounts of closed‐shell cations were found to be synthetically very useful (Scheme 4 D). The pioneering work of Fukuzumi et al.^[56]^ paved the way for many publications based on the use of acridinium‐based donor–acceptor dyads as strongly oxidizing photocatalysts.[^17^, ^57^, ^58^, ^59^, ^60^] Moreover, a new benchmark regarding the excited‐state potential was set by using pyrylium, quinolinium, or diazapyrenium salts as extremely powerful photooxidants.^[17]^ Among other cationic dyes, the photoexcited pyrylium or acridinium salts (e.g. **TPT^+^** and **ACR^+^**, Figure 2) are strong oxidants in their excited states and have found widespread synthetic applications.[^17^, ^61^, ^62^, ^63^, ^64^, ^65^, ^66^, ^67^, ^68^] Surprisingly, in contrast to the wealth of reports dealing with photoexcited cations, the photochemistry of closed‐shell anions has received far less attention, although it constitutes the logical counterpart (Scheme 4 E).

Hence, in the following section the ability of anionic photocatalysts to drive challenging transformations is underlined through selected examples. As a consequence of their moderate redox potentials and the wealth of available reviews, reactions of anionic xanthene dyes such as eosin Y, rose Bengal, or fluorescein are not discussed herein.[^17^, ^69^, ^70^, ^71^] Furthermore, examples where anionic groups are mainly installed to increase the solubility of the sensitizer (e.g. 9,10‐anthraquionone sulfonate salts) in polar media without changing its reactivity in a significant manner are excluded.

### 2.2 Phenolate‐Catalyzed Oxyarylation of Olefins with Aryl Halides

**Scheme 5:**
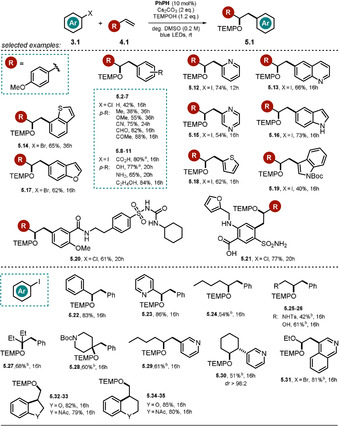
Scope of the oxyarylation reaction of olefins with aryl halides and TEMPOH. [a] With 3 equiv Cs_2_CO_3_. [b] With 3 equiv olefin.

**Scheme 6:**
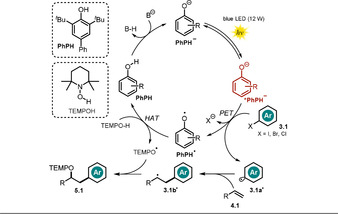
Proposed mechanism for the phenolate‐catalyzed oxyarylation of olefins via the generation of aryl radicals.

The low p*K*_a_ value of phenol, caused by the charge‐stabilizing effect of the benzene ring, allows facile deprotonation in the presence of base to afford the phenolate, which is able to undergo photochemical reactions under irradiation with visible light. Xia and co‐workers examined several 4‐phenylphenol derivatives as potential photocatalysts for the oxyarylation of olefins upon the photoreduction of aryl halides initiated by visible light (Scheme 5).^[72]^ 4‐Phenylphenol bearing bulky *tert*‐butyl groups adjacent to the phenolic alcohol (Scheme 6) showed the highest catalytic efficiency, and the corresponding oxyarylated products **5** formed in the presence of aryl halides **3**, olefins **4**, and TEMPOH could be isolated in moderate to good yields. Remarkably, the estimated excited‐state oxidation potential of *PhPH^−^ ($E_{ox}{}^{*}$ =−3.16 V vs. SCE) also allowed more inert and electron‐rich aryl bromides and chlorides to be converted in the presence of 4‐methoxystyrene. The developed procedure showed a broad scope, tolerating (hetero)aryl bromides and iodides including polyaromatic hydrocarbons, pyridines, indoles, quinolines, thiophen, thianaphthene, and benzofuran.

Various styrenes, aliphatic olefins, allylic sulfonamide and alcohol derivatives, enol ethers, as well as 1,1‐ and 1,2‐ disubstituted olefins were tolerated in the reaction. In addition, the method enabled intramolecular cyclization reactions using aryl iodides and the late‐stage modification of pharmaceuticals. Noteworthy, the use of TEMPOH as a H‐atom donor and radical trap seems to be crucial because of the weak nature of the O−H bond and the high stability of the aminoxyl radical formed. The proposed reaction mechanism involves the deprotonation of the phenol **PhPH** by base and PET from the photoexcited *PhPH^−^ to the aryl halide **3.1**. Upon cleavage of the halide anion, the resulting aryl radical is trapped by the olefin **4.1**, thereby resulting in a carbon‐centered radical **3.1 b^.^**. Hydrogen atom transfer between the oxidized species of the catalyst and TEMPOH recovers **PhPH** and leads to the stable radical TEMPO^.^. The oxyarylation product **5.1** is formed upon radical–radical coupling (Scheme 6). The formation of a ground‐state electron‐donor‐acceptor complex (EDA) between the phenolate anion and aryl halide was excluded by UV/Vis measurements. Fluorescence quenching experiments and isolated TEMPO‐trapping adducts of the aryl radical intermediate support the mechanistic hypothesis. Moreover, a radical clock experiment suggests the formation of a benzylic radical, whereas intramolecular trapping experiments disprove the involvement of a benzylic carbocation formed upon oxidation of the radical **3.1 b** ^.^.

### 2.3 Naphtholate‐Catalyzed Dehalogenation and Detosylation

**Scheme 7:**
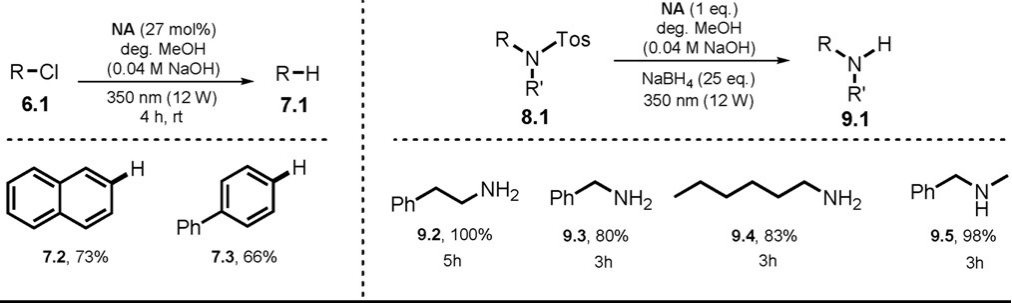
Scope of the **NA^−^**‐catalyzed dechlorination (left) and desulfonylation reactions (right).

The first studies on the photochemical behavior of 2‐naphtholate anion **NA^−^** date back to 1989, when the countercation, temperature, and solvent were systematically evaluated for their effects on the luminescence lifetime and the absorption and emission maxima.^[10]^ In the same year, Soumillion et al. demonstrated the application of the naphtholate anion in the photocatalyzed defunctionalization of 2‐chloronaphthalene and 4‐chlorobiphenyl (**6.2**, **6.3**) in degassed, alkaline MeOH (Scheme 7, left).^[73]^

**Scheme 8:**
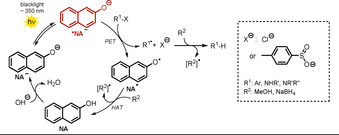
Proposed photocatalytic cycle for the naphtholate anion.

This concept was further extended in a heterogeneous approach where 2‐hydroxynaphthoic acid was covalently anchored to a silica surface through an amidation reaction. The efficiency of the dichlorination, however, was significantly decreased.^[74]^ The substrate scope was later broadened to mono‐ and dichloronitrobenzenes.^[75]^ In addition, **NA^−^** was shown to catalyze the detosylation of sulfonamides in the presence of excess NaBH_4_ as the terminal reductant (Scheme 7, right).^[76]^ Following this procedure, 2‐phenylethylamine (**9.2**) and *N*‐methylbenzylamine (**9.5**) were obtained in quantitative yield starting from the respective sulfonamides. Although a stoichiometric amount of 2‐naphthol (**NA**) was utilized, the catalyst could be efficiently regenerated. The proposed reaction mechanism suggests the deprotonation of **NA** to form the naphtholate **NA^−^**. Upon excitation with black light, the photoexcited state of *NA^−^ is oxidatively quenched by either aryl chloride or sulfonamide, which causes the formation of **NA^.^** and an arene radical anion. After cleavage of the respective anionic leaving group (Cl^−^ or 4‐Me(C_6_H_4_)SO_2_^−^), either an aryl‐ or nitrogen‐centered radical is formed. Abstraction of a hydrogen atom from the solvent affords the defunctionalized arene. The N‐centered radical converts into the amine through H‐atom abstraction from either the solvent or NaBH_4_. To close the catalytic cycle, **NA^.^** is transformed into **NA** through hydrogen atom abstraction from the solvent or NaBH_4_, followed by subsequent deprotonation (Scheme 8).

**Scheme 9:**
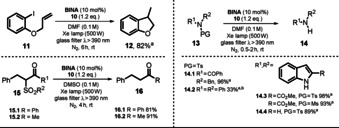
Cyclization of iodoarene and scope of the desulfonylation. [a] NMR yields. [b] DMSO, 6 h.

**Scheme 10:**
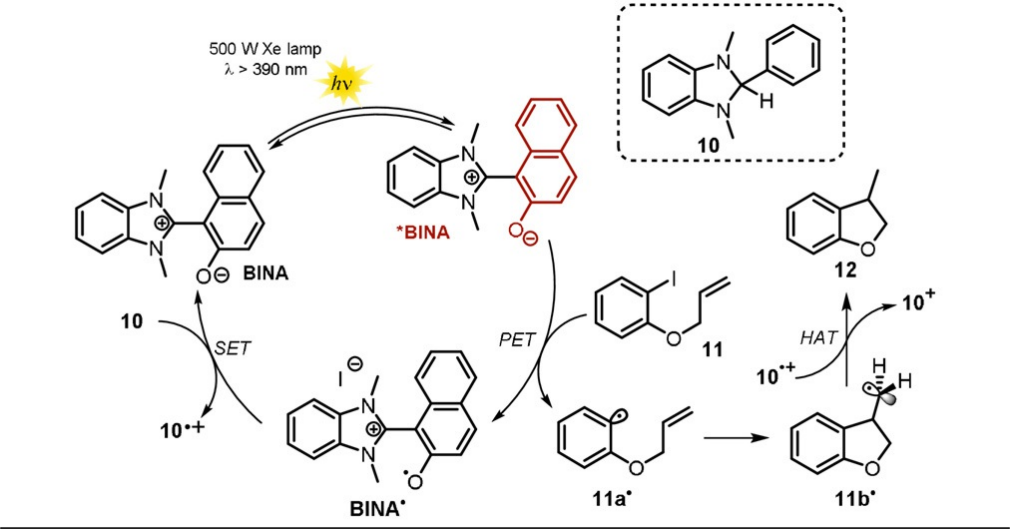
Proposed catalytic cycle for the radical cyclization of iodoarene in the presence of photoexcited benzimidazolium naphtholate.

Recently, a zwitterionic visible‐light‐absorbing benzimidazolium naphtholate **BINA** was successfully employed in photocatalytic deiodination and desulfonylation reactions in the presence of a combined electron and hydrogen atom donor **10** (see Scheme 10).^[77]^ The cationic benzimidazolium moiety can be considered as separated from the naphtholate, since the tilted structure prevents π‐conjugation. The photocatalytic activity was studied using different solvents with attributed Lewis‐basic or Lewis‐acidic characters, as estimated by donor and acceptor numbers. The authors concluded that Lewis‐basic solvents cause tight interactions with the Lewis‐acidic benzimidazolium moiety, whereas the electronic properties of the Lewis‐basic naphtholate anion are less governed, thereby resulting in an increased electron‐donating ability. The best results (Scheme 9) were found using DMF as solvent. Utilizing 10 mol % of catalyst **BINA** and 1.2 equiv of **10** enabled the formation of cyclized **12** in 82 % yield. A lower catalyst loading of only 1 mol % resulted in full conversion of the iodoarene **11**; however, the product yield was lowered (69 %). In addition to the cyclization of iodoarene, the photocatalytic reactivity was demonstrated through the reductive desulfonylation of tertiary sulfonamides **13** and β‐ketosulfones **15**. The respective secondary amines and desulfonylated ketones were obtained in good yields. The proposed photocatalytic cycle is depicted in Scheme 10.

Upon photoexcitation (λ>390 nm), the zwitterionic excited‐state catalyst *BINA ($E_{ox}{}^{*}$ =−2.08 vs. SCE) reduces **11** through PET. Subsequent cleavage of iodide followed by fast 5‐*exo*‐*trig* cyclization affords the primary radical **11 b^.^**. The oxidized photocatalyst **BINA^.^** is regenerated in the presence of a sacrificial reductant **10** (*E*_1/2_=+0.34 V vs. SCE) by single‐electron transfer to give the radical cation **10^.+^**, which acts as the hydrogen atom donor to form **12** and in turn is converted into the cation **10^+^**. In the presence of other terminal reductants, for example, the Hantzsch ester (*E*_1/2_=+0.93 V vs. SCE), no product was formed as the higher oxidation potential of the ground state renders an electron transfer towards **BINA^.^** endergonic.

**Scheme 11:**
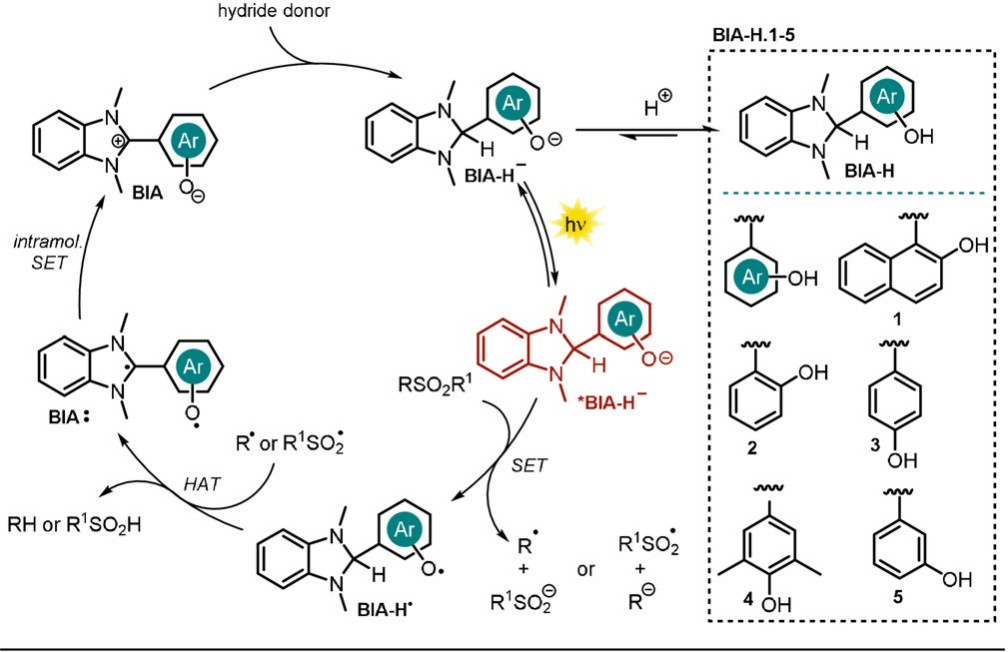
Proposed photocatalytic cycle for the desulfonylation reported by Hasegawa et al.

In previously published work, photoexcited 1,3‐dimethyl‐2‐hydroxynaphthylbenzimidazoline (**BIA‐H.1**) was found to convert *N*‐sulfonamides and *N*‐sulfonylamines into the respective desulfonylated products.^[78]^ Based on these results, Hasegawa et al. further developed the catalytic system depicted in Scheme 10 by utilizing the in situ reduction of benzimidazolium aryloxides (**BIA**) in the presence of readily available boron hydride donors to generate the anionic species **BIA‐H^−^** (Scheme 11).^[79]^

**Scheme 12:**
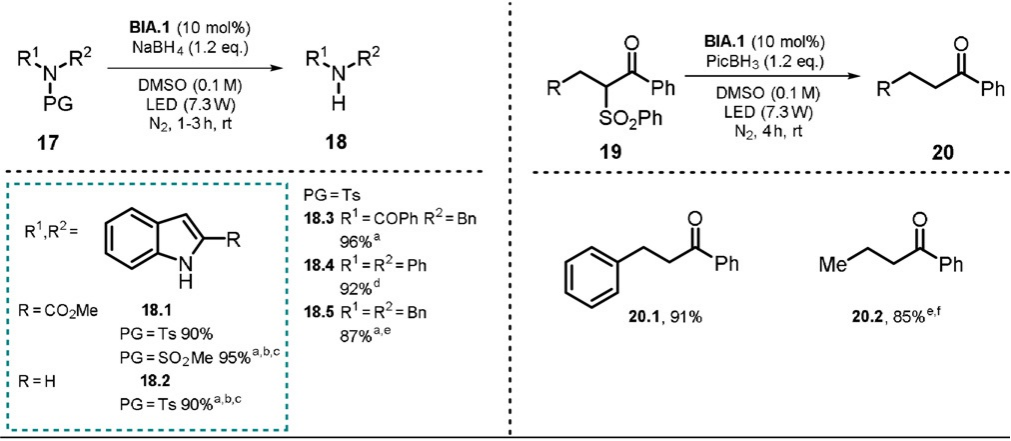
Scope of the desulfonylation. [a] NMR yield. [b^]^ DMF. [c] Xe lamp (500 W), glass filter *λ*>390 nm;. [d] 24 h. [e] 2×LED (10.8 W), 48 h. [f] Cs_2_CO_3_ (1 equiv).

In addition to the reported electron‐donor and hydrogen atom donor abilities of the benzimidazoline scaffold (see Scheme 10, **10**), the resulting benzimidazoline aryloxides **BIA‐H^−^** are equipped with a photoredox active unit, the aryloxide moiety. Reductant, H‐atom donor, and photocatalyst are thus combined in one molecule. Various benzimidazoline aryloxides **BIA‐H.1**–**5** (Scheme 11) were synthesized and characterized in terms of their spectroscopic and electronic properties.^[79]^ The calculated excited‐state oxidation potential for **BIA‐H.1^−^** ($E_{ox}{}^{*}$ =−2.71 V vs. SCE) was found to be significantly enhanced compared to the zwitterionic species **BINA**, thus allowing the conversion of less activated substrates. The elaborated procedure was used for the reductive desulfonylation of *N*‐sulfonylindoles, ‐amides, ‐amines, and α‐sulfonyl ketones to afford the unprotected secondary amines as well as the α‐defunctionalized ketones in good to excellent yield (Scheme 12).

For the desulfonylation of α‐carbonyl compounds, the less reactive hydride donor PicBH_3_ was used to avoid the direct reduction of the carbonyl group. Remarkably, utilizing the developed photocatalytic procedure allowed diphenylsulfonamide (**17.4**) and dibenzylsulfonamide (**17.5**) to be converted almost quantitatively in 24 and 48 hours, respectively. Note that both substrates exhibit a challenging reduction potential (*E*_1/2_<−2 V vs. SCE). All synthesized catalysts **BIA.1**–**5** were successfully tested in the desulfonylation reaction of *N*‐tosylindole **17.1**, but **BIA.1** (or **BINA**, see Scheme 10) showed superior catalytic activity. Changing the light source from a xenon lamp (500 W, λ>390 nm) to a white LED (7.3 W) afforded comparable product yields, but the reaction time increased. No product was formed in the absence of photocatalyst and only traces were found in the absence of a hydride donor or light. In terms of the mechanism, the authors propose the in situ formation of **BIA‐H^−^** through nucleophilic attack of a hydride on the benzimidazolium moiety of **BIA**. Excitation with either a Xe lamp or a white LED renders the catalyst a strong photoreductant and allows PET to the substrate. The open‐shell fragment formed upon rupture of a N−S or C−S bond abstracts a hydrogen atom from the photocatalyst **BIA‐H^.^**, which is turned into a biradical **BIA^.^**. The benzimidazolium **BIA** is regenerated upon intramolecular single‐electron transfer. Eventually, a hydride transfer activates the catalyst for another catalytic cycle (Scheme 11). The acidic hydroxy group on the aryl oxide is easily deprotonated and enables the benzimidazoline **BIA‐H** to be employed directly instead of the betaine **BIA** as the catalyst. In that case, the addition of base (sodium carbonate or butoxide) increased the reaction efficiency significantly, thus indicating a facile deprotonation of **BIA‐H**.

### 2.4 Anthrolate‐Catalyzed Generation of Hydrated Electrons

**Scheme 13:**
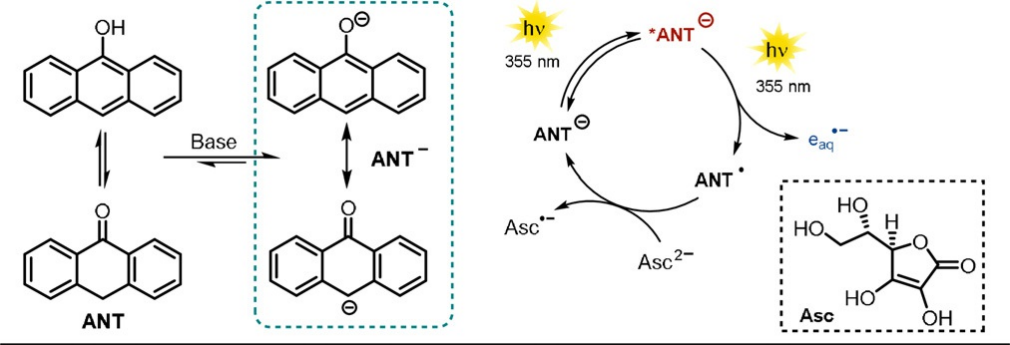
The photocatalytic generation of hydrated electrons reported by Goez and co‐workers.

Kerzig and Goez thoroughly investigated the potential use of anionic 9‐anthrolate (**ANT^−^**) as a sustainable source for hydrated electrons, which are ejected upon irradiation with a laser.^[20]^ Hydrated electrons are among the strongest reductants[^80^, ^81^, ^82^] and are capable of reducing dinitrogen^[83]^ or carbon dioxide directly.^[84]^ Approaches to liberate solvated electrons photochemically often rely on highly energetic and harmful UV‐C light. Notably, irradiation of **ANT^−^** in alkaline aqueous media with a pulsed UV‐A laser (355 nm) afforded hydrated electrons through a biphotonic photoionization pathway. The first photon generates the excited anionic species (S_1_ state) and the absorption of another photon within the excited‐state lifetime of *ANT^−^ stimulates photoejection of a hydrated electron. The catalytic cycle is closed in the presence of the ascorbate dianion **Asc^2−^**, which acts as a sacrificial reductant to recover the catalyst from its oxidized species **ANT^.^** (Scheme 13). The sequence of photoionization and regeneration of the catalyst could be repeated several times until the system was exhausted. At the same time, the initial concentration of the catalyst remained constant, indicating the robustness of anthrolate against an attack of the exceptionally reducing solvated electron. Despite its minute molar absorption coefficient at the wavelength used for exciting the system, **Asc^2−^** was found to slightly contribute to the generation of hydrated electrons.

A follow‐up study by the Goez group^[21]^ focused on the direct photoionization of **Asc^2−^** in the absence of a catalyst by applying a 355 nm laser pulse. A possible application of solvated electrons generated in this way was demonstrated through the efficient dechlorination of chloroacetate as a generic pollutant in wastewater.

### 2.5 Activation of Aryl Chlorides with 9‐Anthrolate

Recently, the photochemical properties and synthetic applications of a series of 9‐anthrone derivatives were studied by König and co‐workers and the corresponding anions were found to reach remarkable oxidation potentials in the excited state.^[22]^

**Figure 3:**
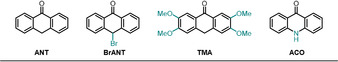
Selected 9‐anthrone‐based photocatalysts reported by König and co‐workers.

In solution, anthrone **ANT** is in equilibrium with its enolic form and is easily deprotonated to give the visible‐light‐absorbing anthrolate **ANT^−^**. The most efficient catalysts examined in that work are depicted in Figure 3.

**Scheme 14:**
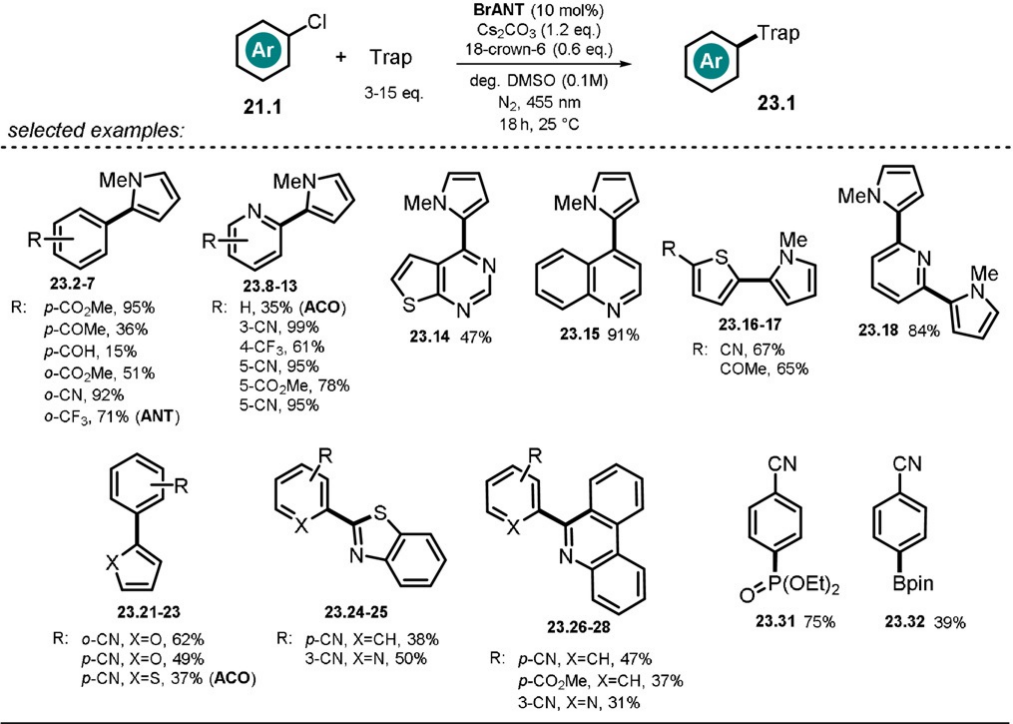
Scope of the C−H arylation of (hetero)arenes using pyrroles, isocyanides, phosphite, and B_2_pin_2_ as trapping reagents. For some substrates, other catalyst derivatives were used, as stated in parenthesis.

**Scheme 15:**
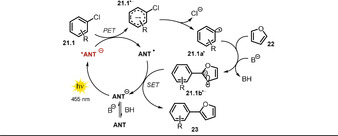
Proposed mechanism for the photocatalyzed C−H arylation in the presence of 9‐anthrone.

These photocatalysts proved successful in catalyzing the C−H arylation of several (hetero)aryl chlorides with electron‐rich (hetero)arenes, isocyanides, phosphite, and B_2_pin_2_ (Scheme 14). In the presence of base, anthrone **ANT** is deprotonated, which causes a red‐shift in the absorption spectrum, together with the appearance of a new distinct absorption band in the visible range. Excitation of **ANT** with blue LED light leads to formation of the strongly reducing excited anion *ANT^−^ (see Table 1, entry 5 for **ANT^−^**). Exceeding the reduction potential of the aryl chloride, oxidative quenching of the excited catalyst would form an arene radical anion **21.1^.−^** and the open‐shell **ANT^.^**. A subsequent mesolytic bond cleavage gives rise to a reactive aryl radical **21.1 a^.^**, which is trapped by an electron‐rich arene **22**. In alkaline media, the emerging bicyclic radical intermediate is deprotonated to afford the radical anion **21.1 b^.−^**. The catalytic cycle is closed through electron transfer from **21.1 b^.−^** to **ANT^.^** (Scheme 15). Time‐resolved luminescence quenching experiments of the excited photocatalyst **ANT^−^** with various tolerated aryl chlorides showed a shortening of the lifetime, whereas unsuccessful aryl chlorides caused no quenching.

TEMPO‐trapping experiments confirmed the formation of aryl radical **21.1 a^.^** and bicyclic radical **21.1 b^.^**. Remarkably, in contrast to other photocatalyzed procedures for the activation of aryl halides,[^38^, ^39^, ^40^, ^41^, ^53^, ^85^] no sacrificial electron donor (e.g. DIPEA) was necessary and the scope of aryl chlorides as the tolerated radical trapping reagents could also be broadened. In the model reaction, the catalyst loading could be lowered to 5 mol % (92 % yield), which indicates a turnover number greater than 18. In accordance with recently reported photocatalyzed C−H arylation procedures,[^38^, ^39^, ^40^, ^41^, ^85^] it was found that an excess of the trapping reagent is crucial for the reaction outcome, as a stoichiometric amount with reference to the aryl halide resulted in a significantly decreased product yield. Anthrolates are converted in the presence of oxygen into the corresponding anthraquinones, thus reactions were carried out under an inert atmosphere. Noteworthy, acridone (**ACO**) afforded the desired arylation product **23.2** in good yield (83 %) in non‐degassed solvent and in the presence of air, thus indicating an increased stability in the presence of oxygen.

### 2.6 Anthrolate‐Catalyzed C−H Carboxylation of (Hetero)arenes and Styrenes with CO_2_

**Scheme 16:**
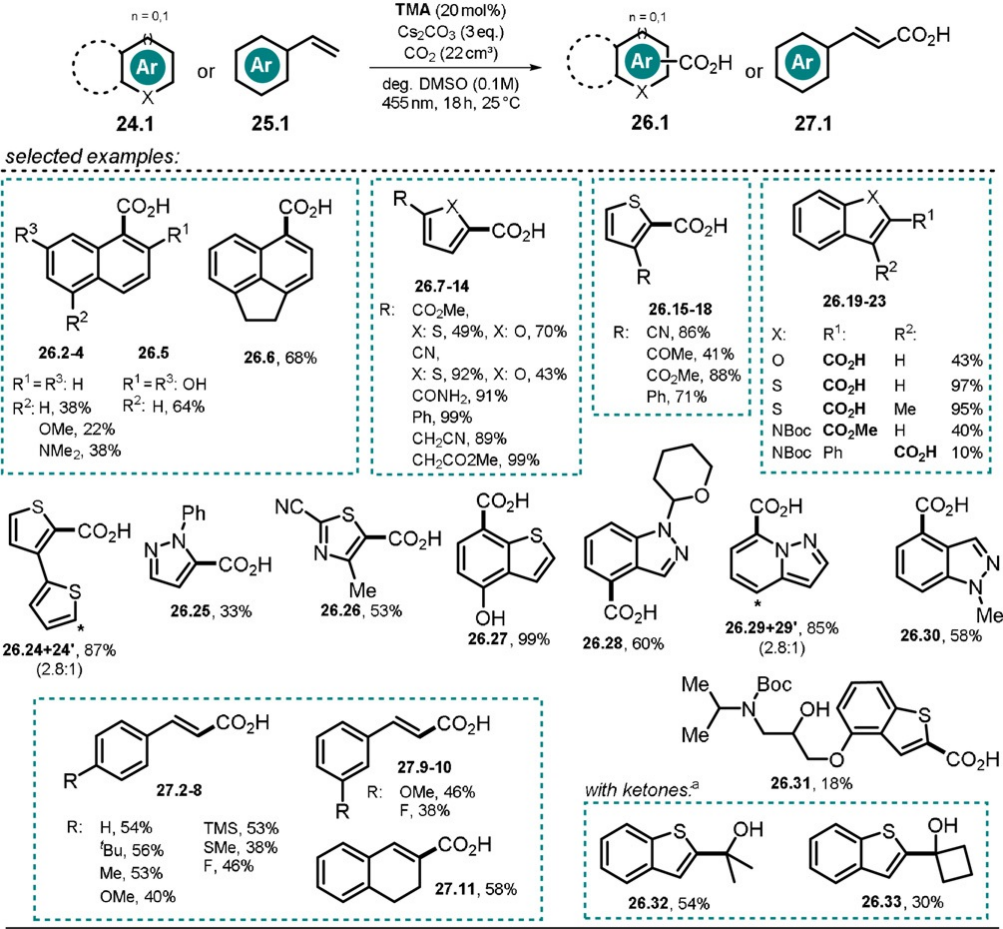
Photocatalyzed C−H carboxylation of (hetero)arenes and styrenes and hydroxyalkylation of thianaphthene. [a] Reaction with ketone (10 equiv) and under a nitrogen atmosphere in the absence of CO_2_.

Very recently, the visible‐light‐absorbing, strong photoreductant tetramethoxyanthrolate **TMA^−^** ($E_{ox}{}^{*}$ =−2.92 V vs. SCE) was utilized to achieve the photocatalytic direct reduction of (hetero)arenes and styrenes to their respective radical anions.^[86]^ The associated nucleophilic character of such electron‐rich species was exploited in C−H carboxylation reactions with gaseous CO_2_ to afford the aromatic carboxylic and cinnamic acids in moderate to excellent yields. Among others, naphthalenes, thiophenes, furans, indoles, pyrazoles, and styrenes that had not been prefunctionalized are converted into the corresponding carboxylic acids under exceptionally mild reaction conditions (Scheme 16). A gram‐scale carboxylation of 2‐cyanothiophene **26.9** illustrates the ease of scaling‐up this reaction. Moreover, a late‐stage C−H carboxylation of a Boc‐protected thiophene analogue of propranolol **26.31** has been demonstrated following this procedure. Besides CO_2_, ketones were found to convert into the corresponding tertiary alcohols (**26.32**, **26.33**) by the same approach. Noteworthy, similar transformations usually require stoichiometric amounts of reactive organolithium reagents and are conducted under low temperature (−78 °C). Thus, a former protection of labile functional groups is often required, thereby leading to a multistep synthesis.[^87^, ^88^, ^89^]

**Scheme 17:**
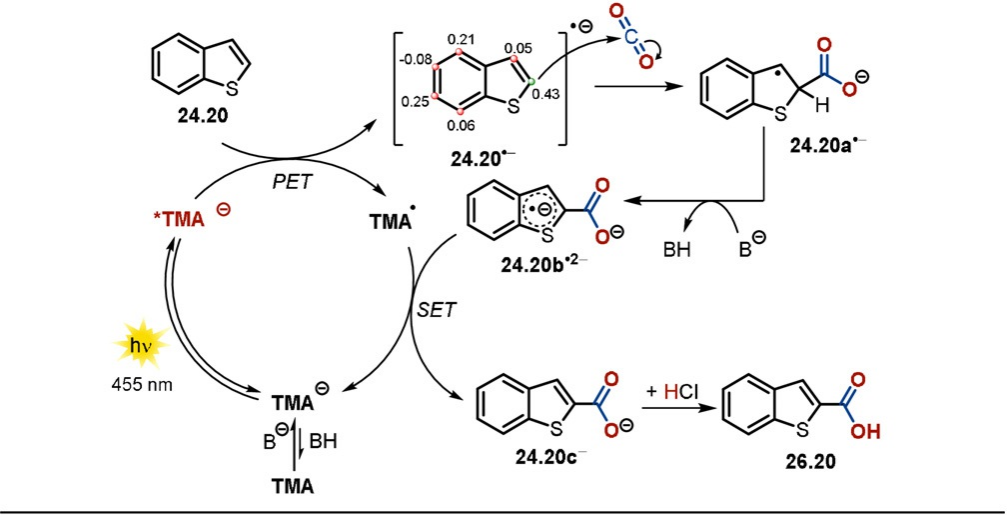
Proposed reaction mechanism for the redox‐neutral, photocatalyzed carboxylation of (hetero)arenes and styrenes utilizing **TMA** as a strong photoreductant. Calculated Mulliken spin populations for **24.20^.−^** allow the regioselectivity of the carboxylation to be predicted.

The regioselectivity of the carboxylation reaction can be predicted by theoretical means. In contrast to the carboxylation mediated by organometallic reagents, the reported photocatalyzed, redox‐neutral insertion of CO_2_ into non‐activated sp^2^‐hybridized C−H bonds benefits from increased regioselectivity, giving rise to only one regioisomer **26.18** and **26.25**, respectively. In the presence of base, **TMA** is in equilibrium with the anionic form which, in contrast to the neutral species, shows distinct absorption in the visible range. Excitation with a blue LED generates the excited state of the anionic catalyst *TMA^−^, which acts as a remarkably strong photoreductant. Upon SET, benzothiophene **24.20** is reduced to the resonance‐stabilized radical anion **24.20^.−^**. Subsequent nucleophilic attack affords the carboxylate **24.20 a^.−^**. The closure of the catalytic cycle is proposed to occur via an electron‐rich radical dianion intermediate **24.20 b^.2−^**, formed upon deprotonation of **24.20 a^.−^**, which regenerates the active anionic catalyst by single‐electron transfer to generate the carboxylate **24.20 c^−^**. Eventually, acidic work‐up affords the carboxylic acid **26.20** (Scheme 17). An alternative pathway by direct H‐atom abstraction from **24.20 a^.−^** by the open‐shell species **TMA^.^** is also conceivable. In both cases, the gain in energy upon rearomatization of the compound is considered as the driving force to close the catalytic cycle.

The mechanistic hypothesis was supported by time‐resolved luminescence quenching experiments of the catalyst *TMA^−^ in the presence of (hetero)arenes and styrenes. Tolerated substrates shortened the excited‐state lifetime of the photocatalyst and linear Stern–Volmer plots could be developed. Although the direct reduction of CO_2_ (*E*_1/2_=−2.21 V vs. SCE)^[90]^ by the excited catalyst is thermodynamically feasible, a DMSO solution saturated with carbon dioxide was found to scarcely affect the excited‐state lifetime. Examined substrates that showed quenching of the photoexcited state of the catalyst but failed to give the respective carboxylic acids are considered to exhibit insufficient nucleophilicity when present as radical anions and thus do not react with carbon dioxide. In addition, deuterium‐labeling experiments of **24.21** in the presence of D_2_O or ^*t*^BuOD showed incorporation of deuterium into the reactive C‐2 position, which supports the assumption of a basic radical anion intermediate.

### 2.7 Catalytic Reactions of Anionic Metal Complexes

**Scheme 18:**
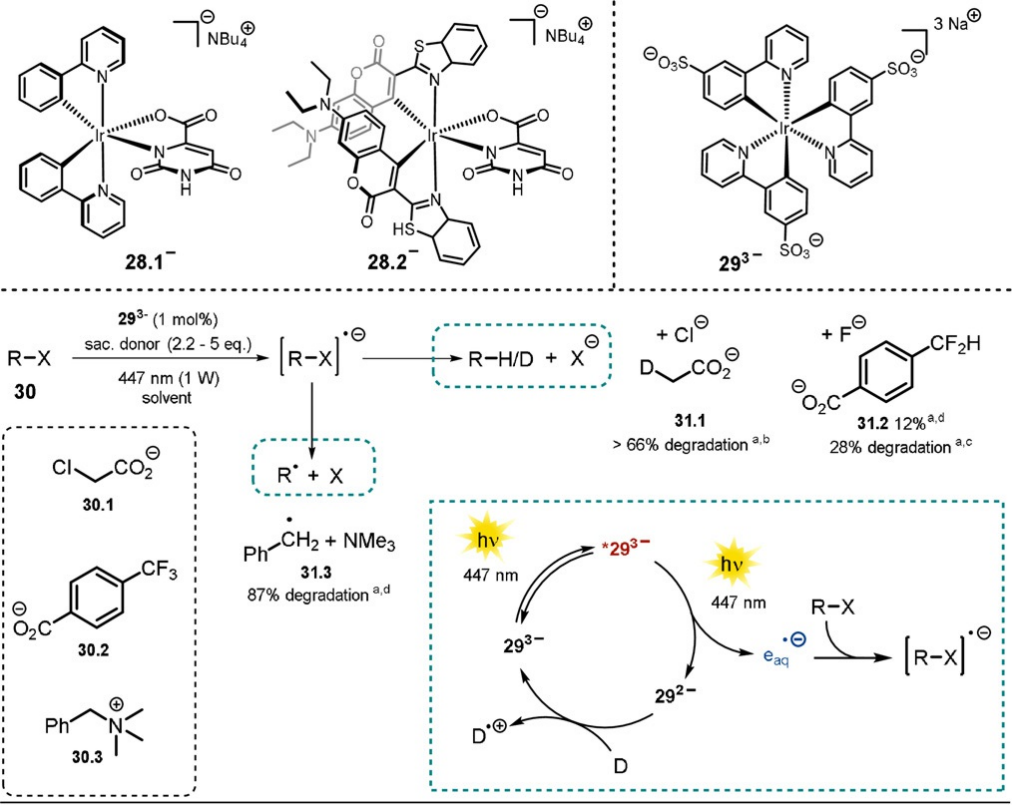
Negatively charged iridium complexes (top); Irsppy (**29^3−^**) catalyzed degradation of pollutants (center); [a] Conversion was determined by NMR analysis of the crude product. [b] Reaction conditions: Chloroacetate **30.1** (12.5 mm), NaHAsc (2.2 equiv) in D_2_O (3 mL), 4 h. [c] Reaction conditions: Trifluormethyl arene **30.2** (15 mm), TEOA (5 equiv) in H_2_O (16 mL), 4 h. [d] Reaction conditions: Benzyltrimethylammonium salt **30.3** (10 mm), TEOA (5 equiv) in D_2_O (3 mL), 3 h. Proposed catalytic cycle for the generation of hydrated electrons (bottom right).

Transition‐metal complexes such as Ru^II^‐polypyridine or the cyclometalated Ir^III^ analogue have found widespread applications in photocatalysis, as they are photostable, show tunable redox potentials, and their excited‐state lifetimes are usually durable. In contrast to neutral complexes, such as *fac*‐Ir(ppy)_3_, or cationic metal‐based sensitizers [e.g. Ru(bpy)_3_^2+^, Ir(ppy)_2_(dtbbpy)^+^], anionic transition‐metal complexes have been barely explored, which could be attributed to their photodecomposition with monodentate anionic ligands^[91]^ and the shortage of available more‐stable dianionic ancillary ligands. Godbert and co‐workers were able to synthesize and characterize the anionic iridium complex **28.1** with a dianionic orotate ligand (Scheme 18, top).^[92]^ Later on, the complex was modified by exchanging the 2‐phenylpyridine ligands with coumarin‐derived ligands (**28.2**) to increase the visible‐light absorption. The authors successfully demonstrated the use of **28.2** in visible‐light‐driven H_2_ generation, which was the first example of a photoinduced electron transfer using an anionic Ir^III^ sensitizer.^[93]^

Based on the well‐established *fac*‐Ir(ppy)_3_, Wenger and co‐workers utilized a trisulfonated analogue **29^3−^** (Scheme 18, top), which renders the sensitizer water‐soluble and negatively charged and generates hydrated electrons.^[94]^ A potential use of hydrated electrons in wastewater treatment was demonstrated by the degradation of chloroacetate **31.1** (Scheme 18) and the benzyltrimethylammonium salt (**31.3**). In addition, the defluorination of trifluoromethylbenzoate is possible in the presence of such a strong reductant (**31.2**). The catalytic cycle is depicted in Scheme 18 (bottom right). The photocatalyst is excited with a 447 nm collimated diode laser. Remarkably, the absorption of a second photon stimulates the ejection of the electron within the lifetime (ca. 1.6 μs) of the excited sensitizer. The photocatalyst is then regenerated by either sodium ascorbate or triethanolamine, which act as sacrificial electron donors. Compared to the neutral *fac*‐Ir(ppy)_3_, the excited‐state oxidation potential of the anionic sensitizer **29^3−^** ($E_{ox}{}^{*}$ =−1.89 V vs. SCE) was found to be slightly increased.

**Scheme 19:**
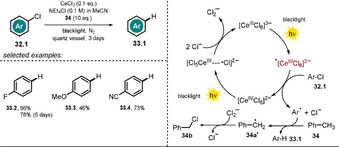
Scope of the CeCl_3_‐catalyzed defunctionalization of aryl halides by the in situ formation of **[Ce^III^Cl_6_]^3−^** and a conceivable mechanism for the reaction.

The trianionic, rare‐earth‐metal catalyst hexachlorocerate(III) **[Ce^III^Cl_6_]^3−^** was found to be effective in the reductive dehalogenation of aryl halides **32.1** using UVA light (Scheme 19).^[95]^ This complex is stable to air and moisture and can be generated in situ by mixing CeCl_3_ and NEt_4_Cl in acetonitrile. Irradiation with black light results in a metal‐centered excited state with a very negative potential ($E_{ox}{}^{*}$ ≈−3 V vs. SCE)[^96^, ^97^] that enables PET to the aryl halide **32.1** to afford a Ce^IV^ species. Interestingly, the reaction could also be performed with a catalytic amount of CeCl_3_, owing to the complementary oxidative photochemistry of **[Ce^IV^Cl_6_]^2−^** (Scheme 19, right).^[98]^ The addition of toluene (**34**) as the terminal reductant allowed to close the catalytic cycle through its conversion into benzyl chloride (**34 b**) upon hydrogen atom abstraction and reaction with Cl_2_^.−^.

**Scheme 20:**
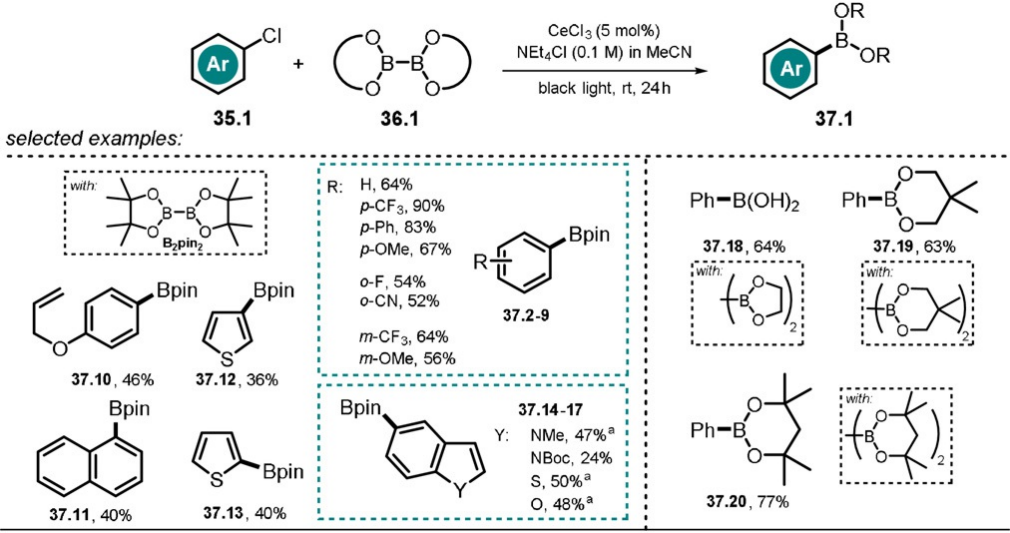
Scope of the **[Ce^III^Cl_6_]^3−^**‐catalyzed Miyaura borylation. [a] Aryl bromide was used.

In a follow‐up study, the developed catalytic procedure was utilized for the photoinduced Miyaura borylation of aryl bromides and chlorides. Schelter and co‐workers used diboron esters which functioned as both the borylation reagent and terminal reductant to close the catalytic cycle.^[99]^ Various arylboronic esters could be obtained in moderate to good yields starting from substituted (hetero)aryl chloride derivatives (Scheme 20). Notably, Stern–Volmer quenching experiments revealed that both electron‐deficient and electron‐rich substrates quench the luminescence of the cerium catalyst. The authors also demonstrated that a sequential borylation and subsequent Pd‐catalyzed cross‐coupling reaction of the formed arylboronic ester is possible. This procedure is beneficial, as it avoids prior isolation of the boronate ester. Based on spectroscopic investigations and experimental findings, a reaction mechanism was proposed (see Scheme 19). The in situ formed **[Ce^III^Cl_6_]^3−^** is photoexcited by black light. Upon PET to aryl chloride **35.1** and loss of Cl^−^, an aryl radical is formed which reacts with the diboron ester **36.1** to yield the aryl boronic ester **37.1** and a boryl radical B(OR_2_)^.^. The oxidized catalyst is regenerated in the presence of excess Cl^−^ through photoinduced ligand‐to‐metal charge transfer, thereby giving rise to the radical anion Cl_2_^.−^. A reaction quantum yield *Φ*>1 was found by actinometry, thus indicating a radical chain mechanism; however, no product formation within the dark periods of an intermittent‐light experiment was observed. The authors consider the boryl radical, which is stabilized in the presence of Cl^−^, to presumably propagate a chain mechanism through reaction with another substrate molecule.

### 2.8 Polyoxometalates as Photocatalysts

**Scheme 21:**
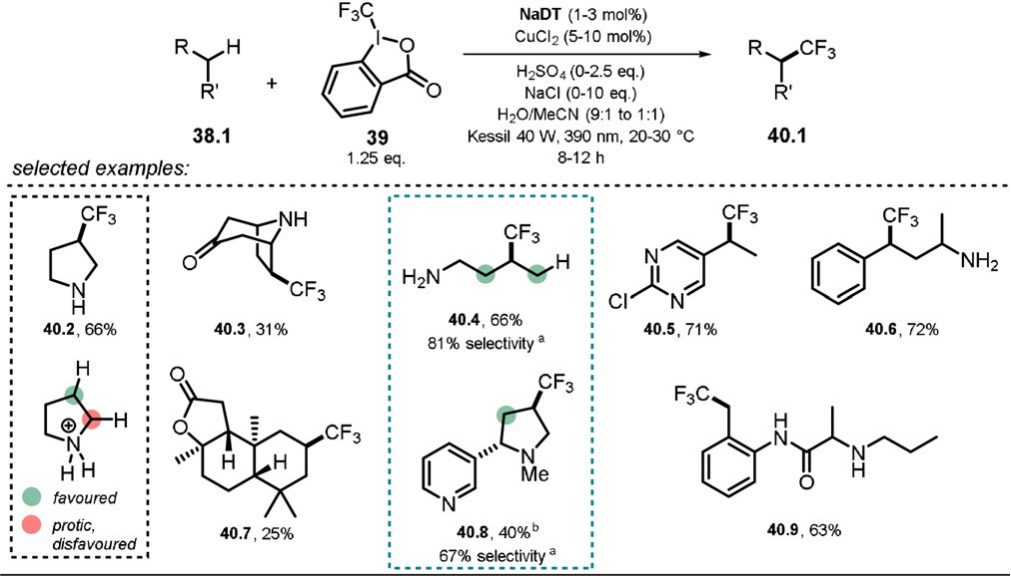
Selected examples of the direct C(sp^3^)−H trifluoromethylation by merging decatungstate catalysis and copper catalysis. [a] The selectivity is reported as the percentage of the major regioisomer over all the regioisomers formed. [b] Major diastereomer shown.

**Scheme 22:**
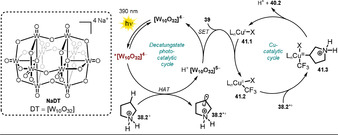
Proposed dual‐catalytic mechanism for direct C(sp^3^)−H trifluoromethylation.

Polyoxometalates (POMs) are a class of widely studied molecular metal oxide anions. Their robustness upon irradiation renders them attractive candidates as catalysts. The discussion of POM photocatalysis will be limited herein to recent, selected examples of decatungstates, which are routinely employed as sodium (**NaDT**) or tetrabutylammonium salts (**TBADT**, see Scheme 22). Hence, for a comprehensive study of POM chemistry we refer the interested reader to excellent reviews.[^100^, ^101^, ^102^, ^103^, ^104^] Despite being negatively charged, these decatungstate anions act as strong oxidants from their excited states. This rare feature might be explained analogously to what was discussed for eosin Y and fluorescein (see Section 2.1). Tungsten is present in its highest oxidation state (+VI), while the negative charge is centered on the oxygen atoms of the cluster, thus rendering the metal center highly electron‐poor and prone to reduction. Upon photoexcitation, a ligand to metal charge transfer (O→M) is proposed to generate a relaxed excited‐state cluster ^*^ **[W_10_O_32_]^4−^** which is easily reduced ($E_{red}{}^{*}$ =+2.44 V vs. SCE).^[100]^ Besides electron‐transfer reactions, excited decatungstate has found widespread interest for its ability to abstract hydrogen atoms from non‐activated C(sp^3^)−H bonds. Fagnoni, Ryu, and co‐workers summarized the site‐selective C−H functionalization of alkanes, alcohols, ethers, ketones, amides, esters, nitriles, and pyridylalkanes by decatungstate and explained the observed regioselectivities on the basis of polar and steric effects.^[105]^ In 2018, MacMillan and co‐workers demonstrated the powerful merger of anionic decatungstate photocatalysis and transition‐metal‐catalyzed cross‐coupling.^[106]^ Based on this concept, a copper/decatungstate dual catalytic approach was recently developed that allowed the C(sp^3^)−H trifluoromethylation of various biorelevant compounds, including natural products and medicinal agents, in moderate to good yield (Scheme 21).^[107]^ The introduction of a CF_3_ group into drug molecules often improves pharmacokinetic properties and is, therefore, of interest. In the case of pyrrolidine (**40.2**), selectivity for the CF_3_ functionalization is achieved upon protonation of the amine, which results in stronger and less hydridic α‐C−H bonds and thus enables reactivity at the distal position. Regioselective functionalization was found at the benzylic (**40.5**, **40.6**, **40.9**) or sterically most accessible, electron‐rich C(sp^3^)−H bond (**40.3**, **40.7**). The reaction is initiated by 390 nm light, which causes an electrophilic oxometallate excited state. Upon hydrogen atom abstraction from the β‐C(sp^3^)−H bond of the protonated pyrrolidinium species **38.2** ^+^, the reduced decatungstate catalyst H^+^ **[W_10_O_32_]^5−^** and the aliphatic radical cation **38.2^.+^** are formed. Subsequent single‐electron transfer to the Togni reagent II **39** regenerates the active HAT catalyst and enables the formation of a copper(II)‐CF_3_ species **41.2**. The pyrrolidinium radical **38.2^.+^** is captured by the copper complex to form an alkyl‐copper(III)‐CF_3_ intermediate **41.3**, and eventually the product **40.2** is formed upon reductive elimination and regeneration of the Cu^I^ catalyst **41.1** (Scheme 22).

**Scheme 23:**
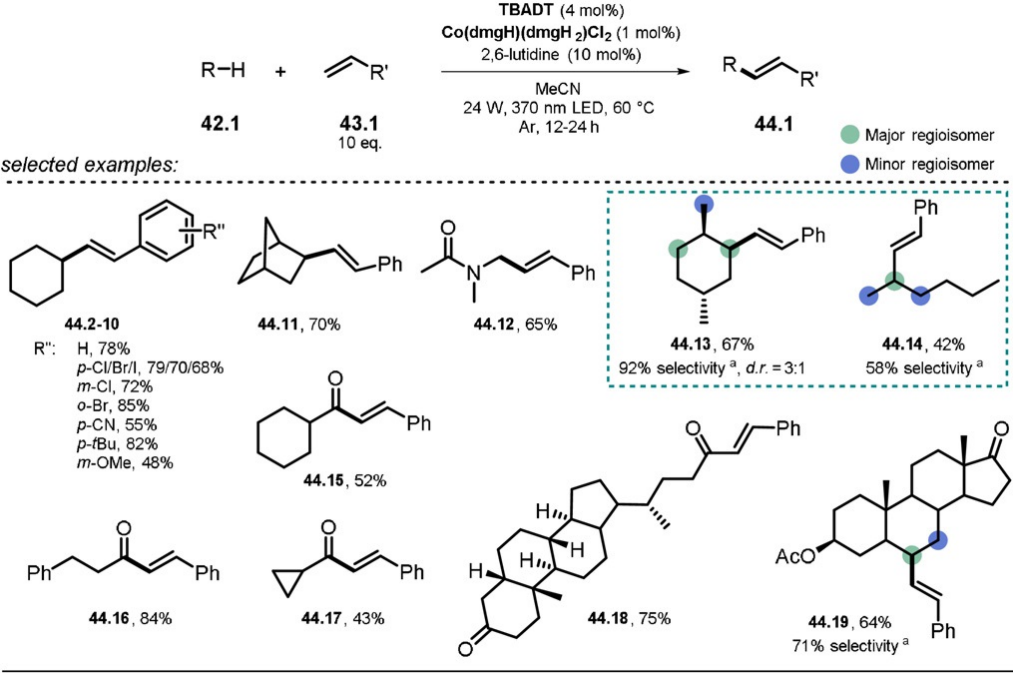
Selected examples of the dehydrogenative alkenylation of alkanes and aldehydes with styrene derivatives. [a] The selectivity is reported as the percentage of the major regioisomer over all the regioisomers formed.

Wu and co‐workers disclosed the oxidant‐free, site‐ and *E*‐selective dehydrogenative alkenylation of alkanes or aldehydes with alkenes by combining decatungstate HAT photocatalysis and cobaloxime catalysis.^[108]^ This dual‐catalytic strategy enables the efficient and direct alkenylation of C−H bonds, with hydrogen gas being the sole by‐product. A broad range of alkanes and aldehydes could be alkenylated. Notably, aryl halides (Cl, Br, I) alkyl bromides, alkenes, and alkynes were tolerated, which enables subsequent orthogonal functionalization through transition‐metal catalysis. Moderate to good regioselectivity was observed for alkane substrates **42.13**, **42.14**, and **42.19**. In addition, the concept could be employed to the late‐stage alkenylation of natural products (Scheme 23).

The excitation of the metal oxide cluster **[W_10_O_32_]^4−^** (**TBADT**) enables the abstraction of a hydrogen atom from alkanes or aldehydes **42.1**. Subsequent addition of the resulting carbon‐centered radical **42.1 a** ^.^ to an alkene **43.1** then furnishes intermediate **42.1 b^.^**. This species is expected to be reversibly captured by the Co^II^ complex **45.1** to form the alkyl‐Co^III^ intermediate **45.2**.

**Scheme 24:**
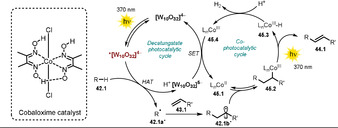
Proposed dual‐catalytic mechanism for the dehydrogenative alkenylation of alkanes and aldehydes with alkenes.

Light‐mediated formal β‐H elimination from **45.2** results in formation of the product **44.1** and Co^III^‐H species **45.3**, which reacts with a proton to release H_2_ and the Co^III^ complex **45.4**. Eventually the decatungstate and the cobalt(II) catalysts are regenerated by SET (Scheme 24).

**Scheme 25:**
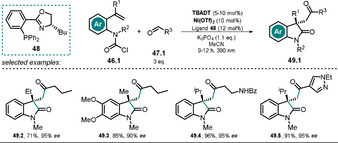
Selected examples of the asymmetric acyl‐carbamoylation.

Wang et al.^[109]^ recently published the **TBADT**/Ni dual‐catalytic asymmetric acyl‐carbamoylation of tethered alkenes by using a chiral nickel catalyst to form oxindole motifs bearing a quaternary stereogenic center **49.2**—**49.5** (Scheme 25).

**Scheme 26:**
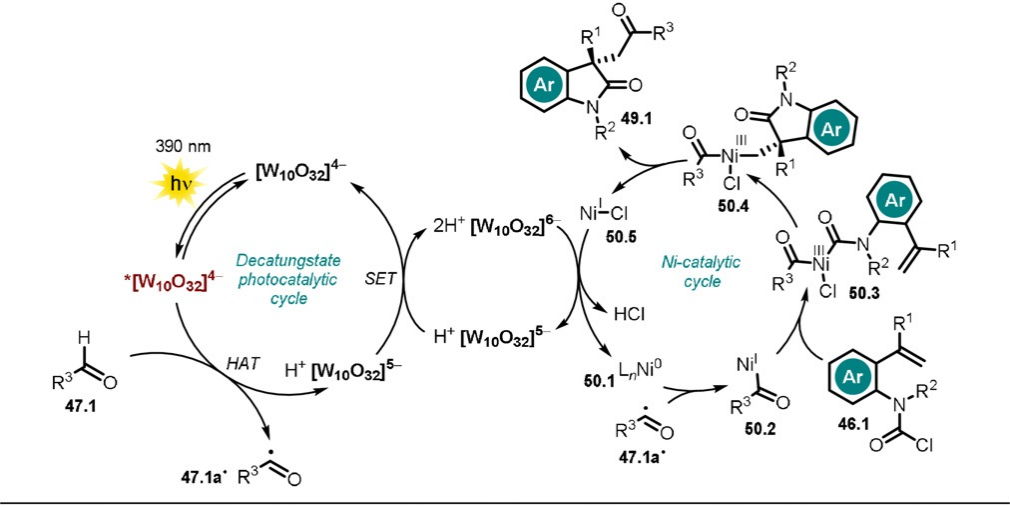
Proposed dual‐catalytic mechanism for asymmetric acylcarbamoylation using a chiral Ni complex.

The reaction starts with H‐atom abstraction from the aldehyde **47.1** by the excited decatungstate catalyst *[W_10_O_32_]^4−^, and the resulting acyl radical **47.1 a^.^** is captured by the in situ formed Ni^0^ to yield an acyl Ni^I^ intermediate **50.2**. Oxidative addition of the carbamoyl chloride **46.1** results in a Ni^III^ species **50.3**. In the enantioselective step, migratory insertion into the tethered double bond takes place (**50.4**) and subsequent reductive elimination affords the cyclized product **49.1** along with Ni^I^ chloride **50.5**. Both catalytic cycles are presumably closed through SET between the reduced decatungstate **[W_10_O_32_]^6−^** and Ni^I^Cl **50.5** (Scheme 26).

Another example of a light‐mediated asymmetric C−H functionalization was recently reported by Pu‐Sheng Wang and co‐workers.^[110]^ Upon hydrogen atom abstraction by **TBADT**, an alkyl, benzyl, or allyl radical adds to an exocyclic enone and the resulting α‐carbonyl radical regenerates the photocatalyst through hydrogen atom transfer. In the enantioselective step, the formed enol intermediate is protonated by an aligned chiral spiro phosphoric acid, thereby generating a stereocenter at the α‐position of the carbonyl moiety.

Based on the synergy of decatungstate HAT catalysis and nickel catalysis, Wang and co‐workers demonstrated the acylation of aryl halides and α‐bromo acetates with aromatic and aliphatic aldehydes, whereby the resulting aromatic ketones and 1,3‐dicarbonyls could be obtained in moderate to good yield.^[111]^ In a similar fashion, the group of Zheng disclosed very recently the direct C−H arylation of aldehydes by merging decatungstate HAT photocatalysis and palladium cross‐coupling catalysis.^[112]^ Application of this method allowed the efficient linkage of various (hetero)aryl bromides, iodides, and triflates with aromatic and aliphatic aldehydes. Moreover, *TBADT was shown to promote H/D exchange reactions of formyl C−H bonds and a wide range of hydridic C(sp^3^)−H bonds in a synergistic system comprised of a HAT photocatalyst and a thiol catalyst. In the presence of D_2_O, this procedure allowed the regioselective incorporation of deuterium into pharmaceutically relevant molecules and drug precursors.^[113]^ Furthermore, a few examples are known where polyoxometalates equipped with binding sites on the cluster shell or in the presence of co‐catalysts participate in the reductive activation of CO_2_ or generation of H_2_.^[35]^

## 3 Excited Anionic Compounds as Reagents

Besides using a light‐harvesting anionic catalyst as demonstrated in Section 2, chemical reactions can also be promoted by a direct photoexcitation of anionic reagents, which will be discussed in the following section.

### 3.1 Excited‐State Phenolate as a Photoreductant

Recently, Xia and co‐workers made use of the remarkable excited‐state potential of the phenolate **52.2** ($E_{ox}{}^{*}$ =−2.48 V vs. SCE) in a Heck‐type arylation reaction promoted by blue LED light.^[114]^

**Scheme 27:**
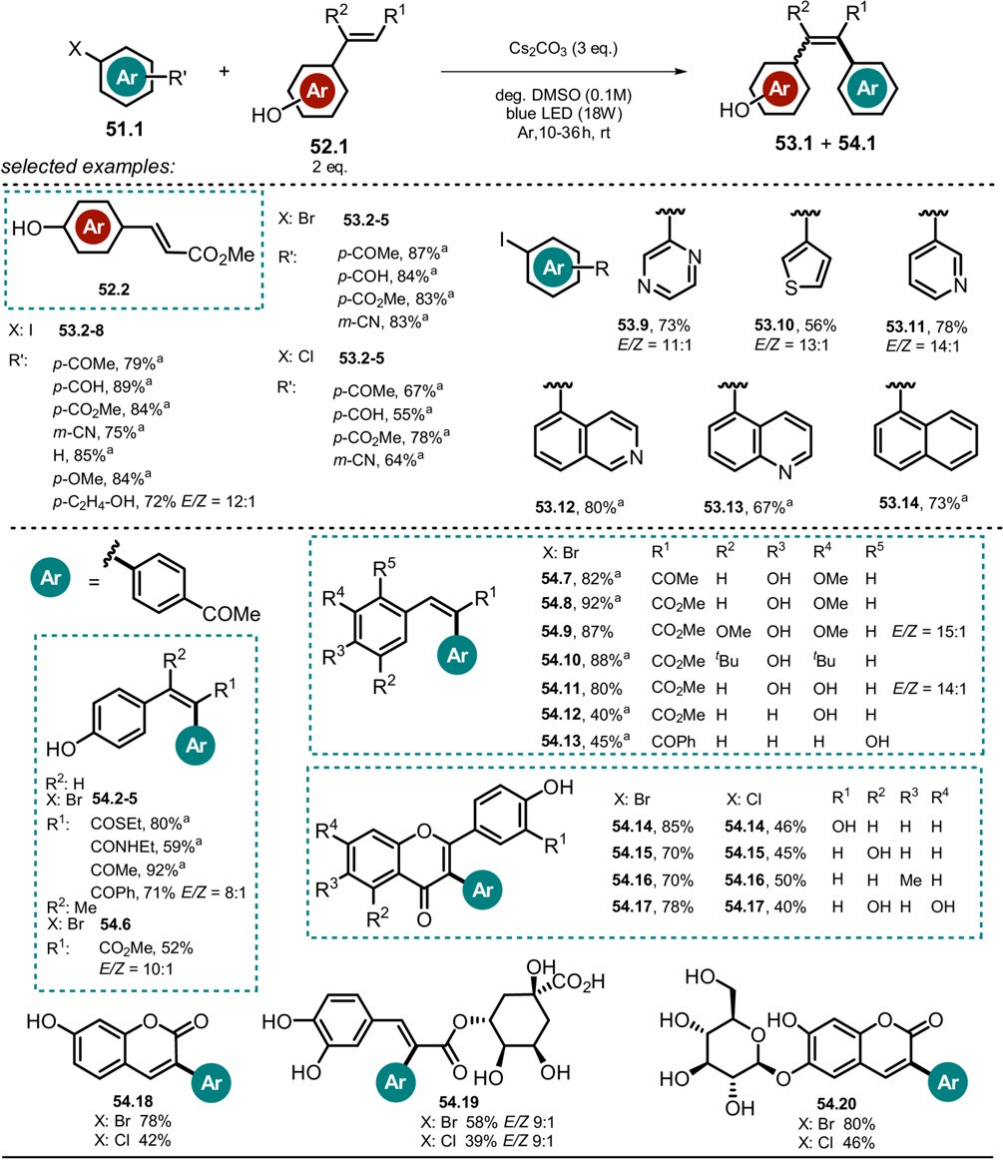
Substrate scope of the Heck‐type arylation reaction reported by Xia and co‐workers. [a] *E*/*Z*>19:1.

**Scheme 28:**
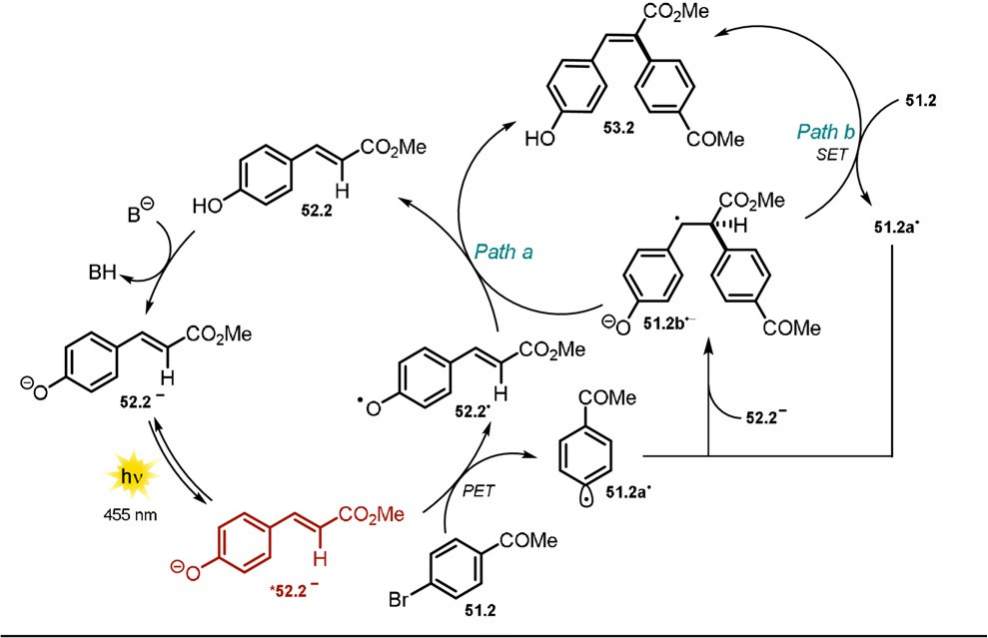
Proposed reaction mechanisms for the photochemical Heck‐type arylation of vinylphenols.

The synthetic utility was demonstrated through the arylation of methyl 4‐hydroxycinnamate (**52.2**) with various (hetero)aryl halides **51.1** (Scheme 27). In addition, other derivatives of cinnamic acid (**52.2**–**52.13**, **52.19**), and flavonoids (**52.16**–**52.18**, **52.20**) were shown to react smoothly via the generated aryl radical to afford the respective arylation products (**53.1** and **54.1**) in moderate to good yields. Remarkably, as the proposed mechanistic cycle is redox‐neutral, no sacrificial electron donor is necessary. Besides electron‐deficient aryl iodides, the scope includes electron‐rich as well as electron‐neutral derivatives. In contrast, arylation products formed with less activated aryl bromides and chlorides are only shown with activated, electron‐deficient arenes. The *E*/*Z* ratios of the formed arylation products are high for most of the isolated compounds. The mild reaction conditions allowed complex, biologically active substrates, such as chlorogenic acid, esculin, and scutellarin, to be converted. Upon deprotonation of the phenolic OH group, the absorption spectrum of **52.2** in DMSO is shifted towards longer wavelength, thereby enabling direct excitation of the phenolate **52.2^−^** with blue light. From the photoexcited state *52.2^−^ (Scheme 28), an electron transfer to the aryl halide **51.2** is feasible and subsequent cleavage of bromide forms the reactive aryl radical **51.2 a^.^**, which preferentially couples to electron‐rich species such as the vinylphenolate **52.2^−^**.

The resulting radical anion **51.2 b^.−^** is assumed to either initiate a radical chain mechanism by reducing another equivalent of **51.2**, which affords the desired Heck‐type arylation product **53.2** (Path b), or is converted into the latter in the presence of the phenoxy radical **52.2^.^** by direct hydrogen atom transfer or electron transfer followed by a proton shift (Path a).

**Scheme 29:**
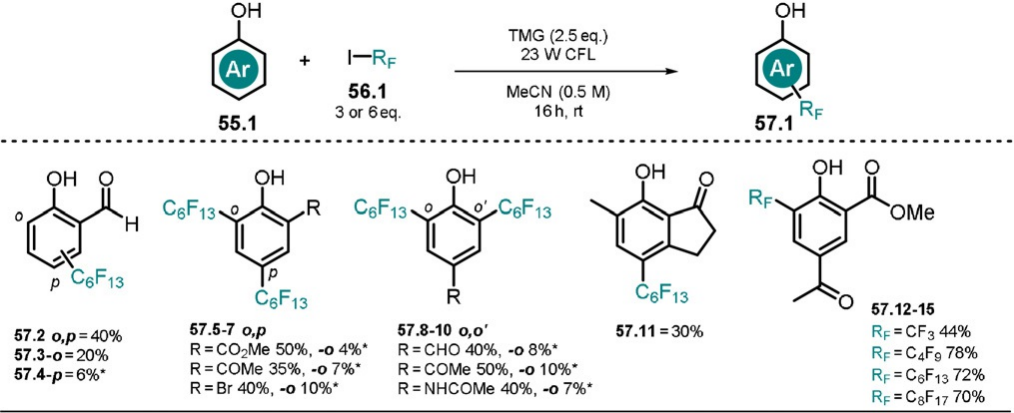
Scope of the perfluoroalkylation of substituted phenols. Minor positional isomers estimated by ^19^F NMR analysis of the crude product are marked with (*); 6 equiv of alkylating agent **56.1** were used for products **57.7**–**57.10**.

**Scheme 30:**
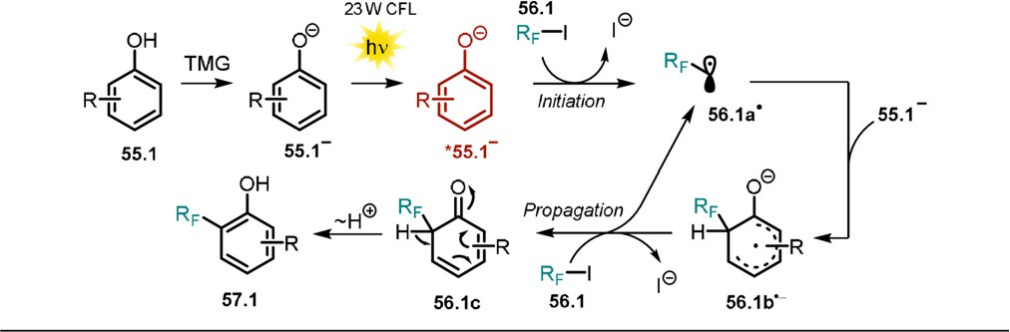
Light‐triggered perfluoroalkylation of phenolates bearing an electron‐withdrawing substituent by Melchiorre and co‐workers. For simplicity, only the *ortho*‐alkylation pathway is shown.

Melchiorre and co‐workers have recently demonstrated how phenolate can elicit the generation of perfluoroalkyl radicals by single‐electron transfer.^[115]^ The developed method allows the direct perfluoroalkylation and trifluoromethylation of phenols bearing electron‐withdrawing substituents **57.2**–**57.15** (Scheme 29). In the presence of the non‐nucleophilic base 1,1,3,3‐tetramethylguanidine (TMG), the absorption spectrum of salicylaldehyde (**55.2**) is red‐shifted and no change was observed upon addition of the perfluoroalkyl iodide **56.1**, thereby excluding the formation of a ground‐state EDA complex. The base‐induced bathochromic shift allowed the use of a compact fluorescent lamp (CFL) as the light source. The use of a 300 W Xe lamp with a cut‐off filter (λ>385 nm) still resulted in the formation of the product, however, in slightly decreased yield. The proposed mechanism of this transformation (Scheme 30) starts with a SET from the photoexcited phenolate *55.1^−^ to **56.1**. Subsequent reductive cleavage of iodine gives rise to a perfluoroalkyl radical **56.1 a^.^**.

In the bond‐forming step, the radical is trapped by the ground‐state phenolate to yield a cyclohexadienyl radical **56.1 b^.−^**, which propagates the reaction by reducing another equivalent of **56.1** by SET. Subsequent proton shift affords the alkylated phenol **57.1**. Stern–Volmer quenching studies of the phenolate in the presence of alkyl iodide support the mechanistic proposal. The perfluoroalkylation proceeds with moderate regioselectivity with *o*‐substituted phenols, giving rise to *o*‐ and *p*‐monoalkylated as well as *o*,*p*‐dialkylated products, whereas *p*‐substituted phenols resulted in the formation of *o*,*o′*‐dialkylated products.

Monitoring the product distribution over the course of the reaction revealed that *o*‐ and *p*‐alkylated products are formed as intermediates and are further converted into bifunctionalized *ortho*‐ and *para*‐adducts. Unsubstituted or methoxy‐substituted phenols as well as nitrophenols failed to convert. Employing phenol **55.12** bearing electron‐withdrawing groups in the *ortho* and *para* position afforded the monoalkylated product as the sole isomer. Perfluoroalkyl iodides with C_8_, C_6_, C_4_, and C_1_ chains can be used in the reaction (**57.12**–**57.15**).

### 3.2 Visible‐Light‐Promoted Arylation of Azaallyl Anions

Chruma and co‐workers demonstrated how irradiation of the colored azaallyl anion **58.1^−^** with visible light notably increases its excited‐state oxidation potential.^[116]^ In the presence of strong bases (p*K_a_*(conjugated acid)>32), the formed 2‐azaallyl anion acts as a super electron donor in the dark^[117]^ and enables the functionalization of non‐activated aryl iodides and tertiary alkyl halides.

**Scheme 31:**
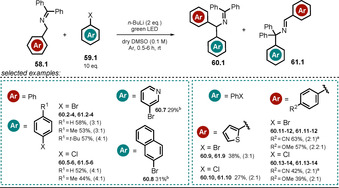
Scope of the light‐mediated azaallyl anion coupling with (hetero)aryl halides. Ratio (**60**/**61**) of the formed regioisomers is given. [a] Blue light. [b] The other regioisomer was not isolated.

The accessible substrate scope could be extended by employing visible light, thereby leading to enhanced reduction potentials that allowed the conversion of non‐activated bromo‐ and chloro‐(hetero)arenes **59.1**, which are present in large excess with respect to **58.1**. The regioselectivity of the arylation reaction is moderate and product mixtures of **60.1** and **61.1** are usually obtained (Scheme 31).

**Scheme 32:**
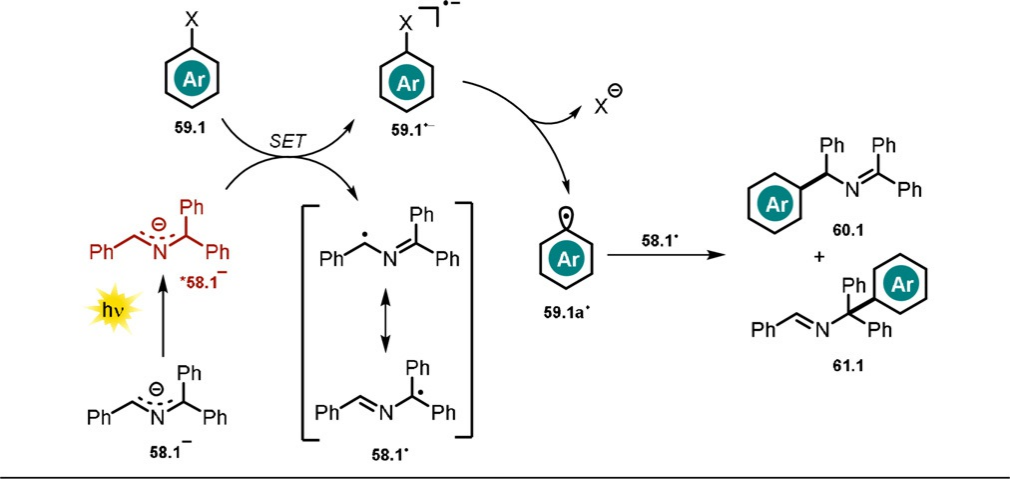
Arylation of 2‐azaallyl anions with non‐activated (hetero)aryl halides.

The authors propose an electron transfer from the excited‐state azaallyl anion ^*^ **58.1^−^** to the aryl halide **59.1**. After cleavage of the carbon–halogen bond, a reactive transient aryl radical **59.1 a^.^** is formed, which reacts with the stabilized azaallyl radical **58.1^.^** to form the arylation products (Scheme 32).

### 3.3 Synthesis of Pyrazoles by Irradiation of α,β‐Unsaturated Hydrazone Anions

**Scheme 33:**
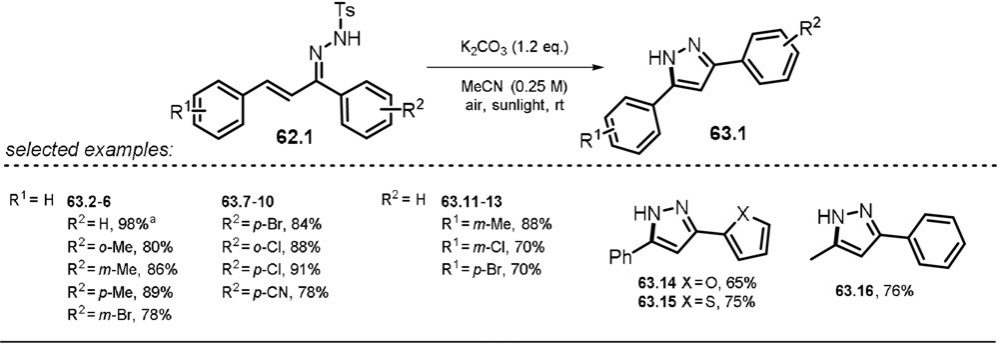
Selected examples of the pyrazoles formed by irradiation of N‐centered hydrazone anions. [a] Scaled up to a 20 mmol reaction.

Zhu and co‐workers reported a series of substituted hydrazones **62.1** which are able to undergo cyclization mediated by sunlight in the presence of base to afford pyrazole derivatives **63.1**.^[118]^ The UV/Vis spectrum of the anionic hydrazone exhibits a significant red‐shift compared to the neutral parent, thereby enabling the use of visible light to accomplish the cyclization reaction. Selected examples of pyrazoles formed are depicted in Scheme 33.

**Scheme 34:**
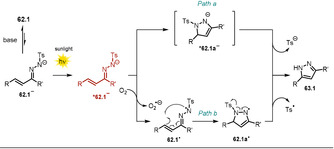
Proposed photoinduced reaction mechanism for pyrazole formation.

The authors propose two possible mechanistic pathways (Scheme 34): Deprotonated **62.1^−^** gets photoexcited and undergoes either direct anionic cyclization to *62.1 a^−^ (Path a) or is oxidized by O_2_ to afford the N‐centered radical **62.1^.^** (Path b), which, upon intramolecular radical cyclization (**62.1 a^.^**) followed by cleavage of a tosyl radical, yields the pyrazole **63.1**. A decreased yield is obtained when the reaction is conducted in a N_2_ atmosphere or in the presence of the radical trap TEMPO, which is indicative of the latter mechanistic proposal (Path b). Notably, the reactions were also shown to operate in water, but resulted in decreased yields.

### 3.4 Utilizing Phthalimide Anions for H‐Atom Abstraction

**Scheme 35:**
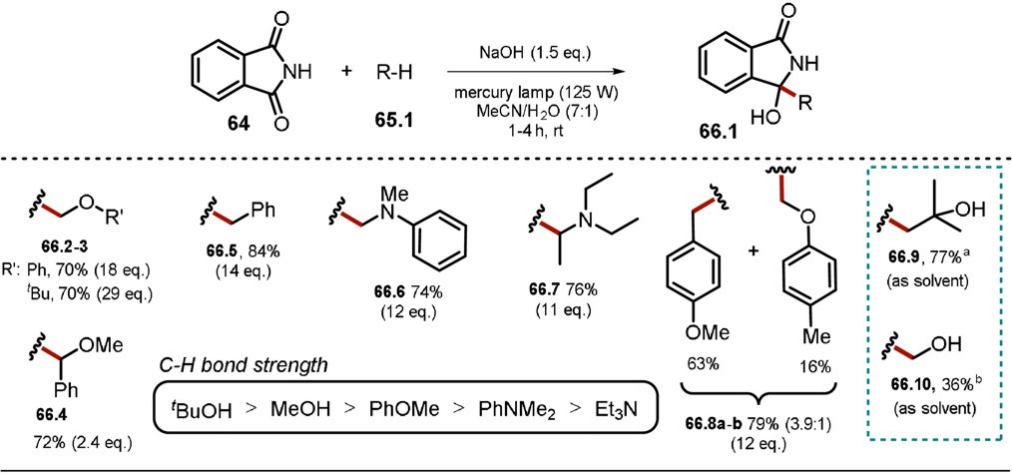
Light‐triggered reductive alkylation of phthalimide. Yields are given based on the consumed phthalimide. Equivalents of the hydrogen atom donor used are given in brackets. Deviation from standard reaction conditions: [a] **64** (13.6 mmol), NaOH (16 mL, 1 m), ^*t*^BuOH (150 mL), mercury lamp (125 W), 5 h. [b] **64** (13.6 mmol), NaOH (10 mL, 1 m), MeOH (160 mL), mercury lamp (125 W), 1 h.

The exceptionally high ability of the excited phthalimide anion *64^−^ to abstract hydrogen atoms from alcoholic solutions was already recognized in 1988.^[119]^ This procedure was further developed and could be extended to ethers, alkylbenzenes, and amines, thereby affording addition products with phthalimide (Scheme 35).^[120]^ The use of 4‐methylanisole afforded a product mixture (**66.8 a**–**b**), as H‐atom abstraction is possible from the methoxy group or the benzylic position. In alkaline solution, phthalimide **64** is in equilibrium with its conjugate base **64^−^**. The photoinduced electron transfer from *64^−^ to ground‐state phthalimide is a thermodynamically favorable process. Thus, the authors propose the phthalimidyl radical **64^.^** as the hydrogen atom abstracting intermediate, which evolves from the excited anion *64^−^ upon PET to phthalimide **64**.

**Scheme 36:**
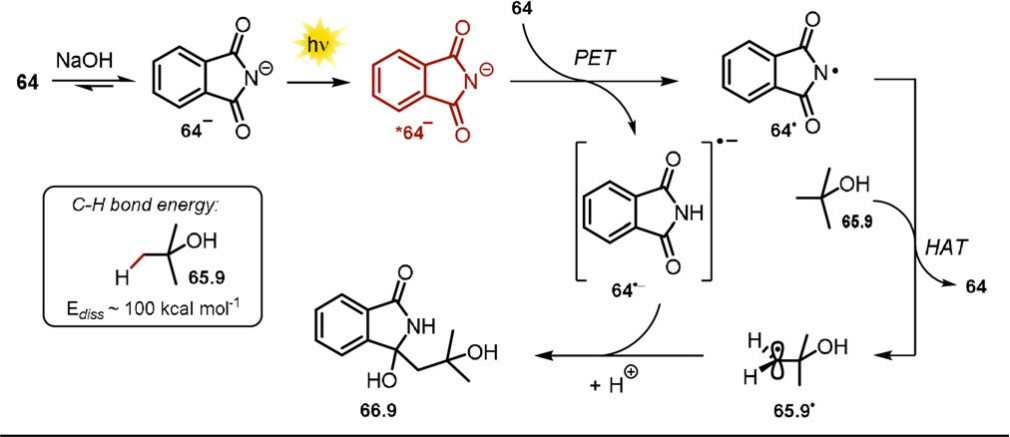
Light‐mediated H‐atom abstraction and radical addition to phthalimide radical anion **64^.−^** initiated by the phtalimide radical **64^.^**.

Remarkably, the electrophilic radical **64^.^** is able to activate C−H bonds possessing high bond dissociation energies (e.g. ^*t*^BuOH, *E*_diss_=100±2 kcal mol^−1^)^[121]^ and upon hydrogen abstraction, phthalimide **64** and the alkyl radical **65.9^.^** are formed. Radical–radical coupling between the phthalimide radical anion **64^.−^** and the carbon‐centered radical **65.9^.^** affords the addition product **66.9** (Scheme 36).

### 3.5 Photocycloadditions of Phthalimide and Saccharin Anions

The formation of [2]benzazepine‐1,5‐dione derivatives **68.1** by [2+2] photocycloaddition using phthalimide **64** was previously limited to electron‐poor noncyclic alkenes, because of competing excited‐state electron‐transfer reactions.[^122^, ^123^]

**Scheme 37:**
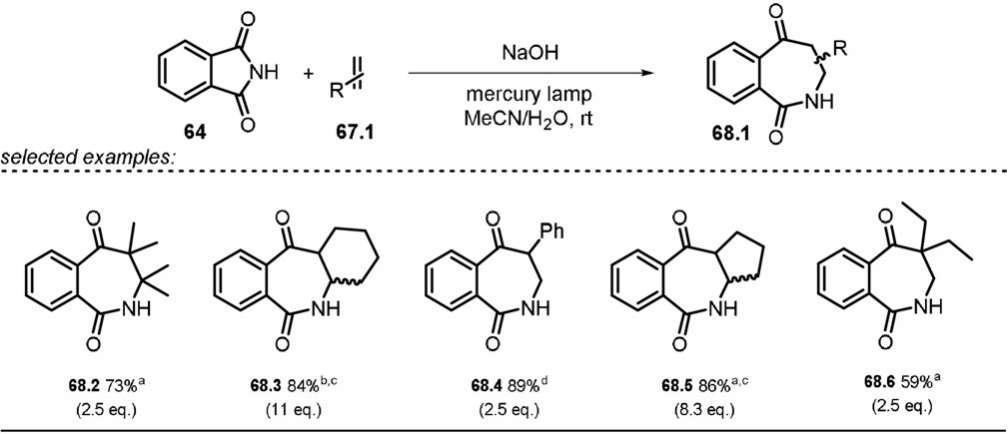
[2+2] photocycloaddition of excited phthalimide anion with alkenes. [a] **64** (6.8 mmol), NaOH (8 mL, 1 m, 1.2 equiv), MeCN/H_2_O (160 mL, 7:1), 0.5 h, 125 W mercury lamp. [b] **64** (6.8 mmol), NaOH (pH≈10), MeCN/H_2_O (7:1), 0.5 h, 125 W mercury lamp. [c] A mixture of *cis*‐ and *trans*‐**68** was obtained. [d] **64** (6.8 mmol), NaOH (10.2 mL, 1 m, 1.5 equiv), MeCN/H_2_O (7:1), 2 h, 400 W mercury lamp.

**Scheme 38:**
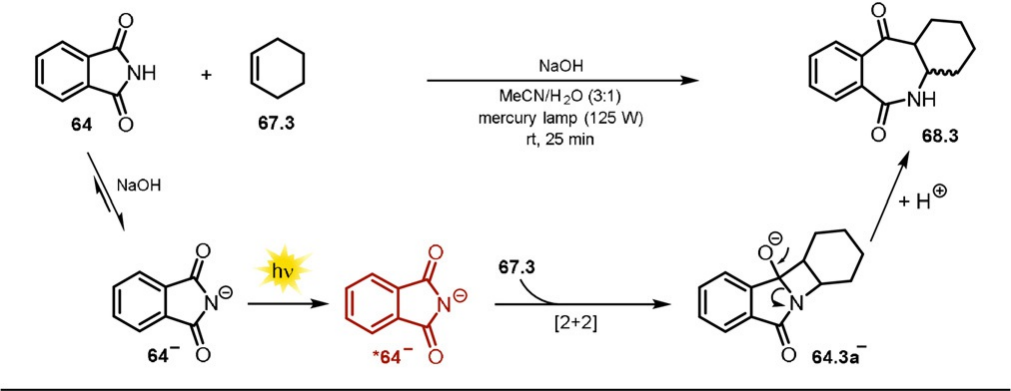
Proposed mechanism for the [2+2] photocycloaddition of the phthalimide anion with cyclohexene.

Suau and co‐workers^[123]^ mitigated the oxidizing strength by employing the anionic sodium phthalimide **64^−^** and obtained efficient, regiocontrolled photocycloaddition and with a broader range of alkenes being tolerated (Scheme 37). In contrast to the neutral species, **64^−^** shows significant fluorescence emission, which was markedly quenched upon alkene addition, which indicates that the singlet excited state *64^−^ is the reactive intermediate. The [2+2] cycloaddition of the photoexcited phthalimide anion to double bonds is a stereospecific process that yields the ring‐expanded *cis*‐**68.1** adduct. Epimerization caused by the alkaline media affords a mixture of *cis*‐ and *trans*‐**68.1** (Scheme 38).

**Scheme 39:**
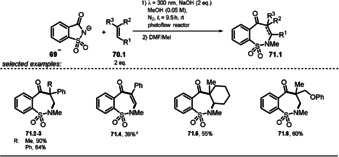
Selected examples of benzosultams isolated upon ring expansion with alkenes. Reaction conditions: Photoflow reactor 0.75 mm internal diameter. [a] Phenylacetylene was used.

The photoexcited saccharin anion *69^−^ was recently found to show similar reactivity towards alkenes, which was utilized in regioselective ring expansion reactions to give benzosultams **71.2**–**71.6** (Scheme 39) starting from the cheap and commercially available sweetener saccharin.^[124]^

**Scheme 40:**
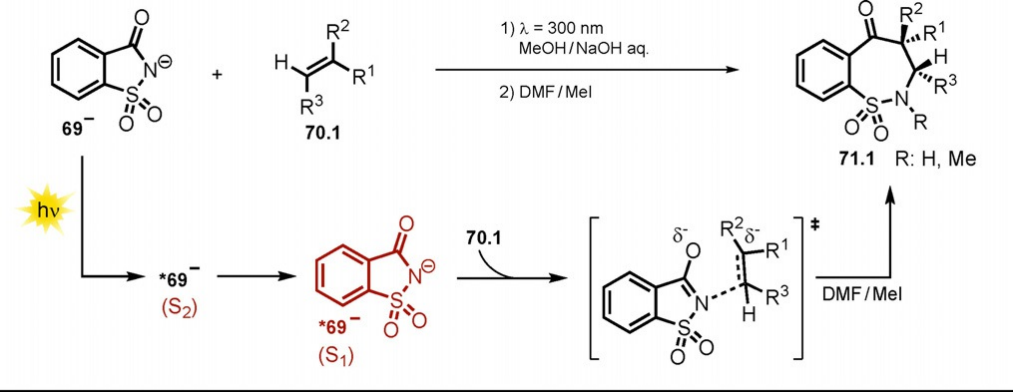
Proposed reaction mechanism for the light‐promoted formation of benzosultams.

Remarkably, common approaches to form benzo‐fused seven‐membered sultam derivatives are multistep reactions and rely on the use of toxic organotin hydrides^[125]^ or expensive Pd catalysts.^[126]^ A mechanism was proposed based on experimental and computational studies that suggests the prevailing population of the S_2_ state upon irradiation of the saccharin anion **69^−^**. The computed data indicate a fast deactivation into the first singlet state. Presumably, the key step towards benzosultam formation is a nucleophilic attack of the nitrogen atom of the excited state saccharin anion at the alkene. Moreover, no evidence for an azetidine intermediate (cf. **64.3 a^−^**, Scheme 38) resulting from the [2+2] cycloaddition of saccharin and alkene was found, neither in experiment nor in computational analysis. The C−C bond formation between the carbonyl group and the alkene is expected to occur in the ground state. Regioselectivity is gained due to the kinetic preference of the nucleophilic nitrogen atom to attack at the terminal, sterically less hindered side (Scheme 40).

### 3.6 Organic Anions Involved in Donor–Acceptor Complexes

Organic anions are also reported to form ground‐state electron‐donor–acceptor (EDA) complexes with electron‐deficient species, and is usually accompanied by the appearance of a new red‐shifted charge‐transfer absorption band. During the last few years, the photochemistry of EDA complexes has become increasingly popular. Among others, we highlight herein three examples to demonstrate the concept of organic anions participating in the formation of an EDA complex. For a more detailed study we refer to recent excellent reviews.[^127^, ^128^] The aromatic perfluoroalkylation of α‐cyano arylacetates **72.1** developed by Melchiorre and co‐workers^[129]^ is mediated by visible light (CFL 23 W), although neither enolate **72.1^−^** nor perfluoroalkyl iodide **56.1** or TMG show absorbance in that range of light. Mixing all the reagents together, however, results in a colored solution with a strong bathochromic shift in the absorption spectrum indicative of the formation of an EDA complex. Irradiation of *p*‐substituted α‐cyano arylacetates in the presence of TMG and an alkylating agent allowed selective perfluoroalkylation at the *ortho* position.

**Scheme 41:**
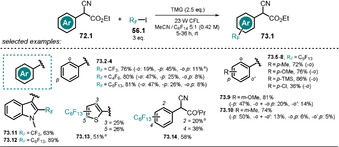
Selected examples of the light‐driven perfluoroalkylation of (hetero)arenes by formation of an EDA complex. [a] Yield determined by ^19^F NMR analysis.

A mixture of regioisomers and dialkylated products was, however, obtained when nonsubstituted or *meta‐ or ortho*‐substituted α‐cyano arylacetates were employed. In accordance with the proposed homolytic aromatic substitution (HAS) pathway, lower yields were obtained with electron‐deficient arenes. Following the developed procedure, the substrate scope could be extended to include heteroarenes (**73.11**–**73.13**) and α‐cyano phenyl ketone **73.14** (Scheme 41). Control experiments revealed that the formed product inhibits the reaction, as the forming enolate **73.1^−^** outperforms the absorbance of the EDA complex. This issue was addressed by utilizing a biphasic system consisting of tetradecafluorohexane and MeCN, which resulted in higher yields and a shorter reaction time.

**Scheme 42:**
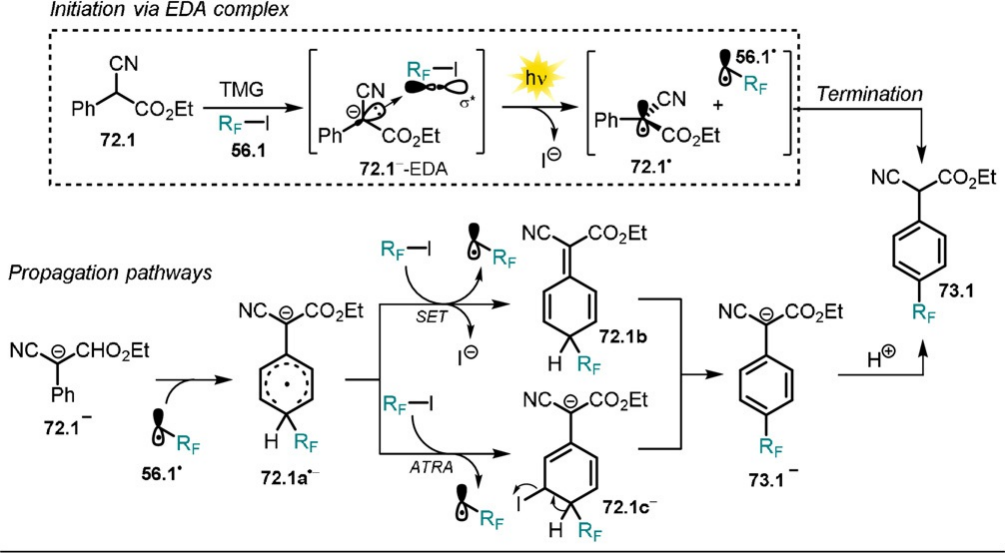
Proposed reaction mechanism for the perfluoroalkylation of α‐cyano arylacetates involving the formation of a visible‐light‐absorbing EDA complex.

The radical chain reaction is initiated by the base‐promoted formation of the EDA complex **72.1^−^**‐EDA, which absorbs visible light and, upon reductive cleavage of iodine, releases a radical pair consisting of the benzylic radical **72.1^.^** and a perfluoroalkyl radical **56.1^.^**. The electron‐rich enolate **72.1^−^** reacts with the alkyl radical through a HAS to afford the radical anion intermediate **72.1 a^.−^**. Chain propagation is assumed either by SET to afford **72.1 b** or by atom‐transfer radical addition (ATRA, **72.1 c^−^**) followed by cleavage of HI. Reaction work‐up yields the perfluoroalkylated product **73.1**. Termination of the radical chain is possible by direct radical–radical coupling of **72.1^.^** and **56.1^.^** (Scheme 42).

**Scheme 43:**
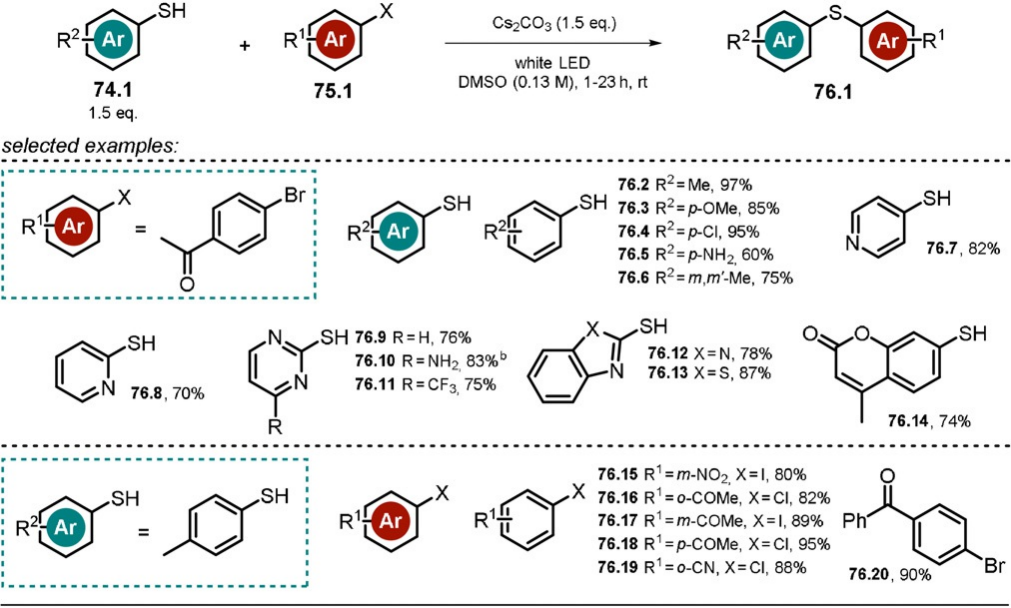
Selected examples of the thiolation of aryl halides. [a] 50 mmol scale. [b] Cs_2_CO_3_ (2 equiv).

The Miyake group made use of the EDA complex formed between an electron‐rich thiolate anion **74.1^−^** and aryl halides **75.1** to afford a broad scope of aromatic thioethers **76.1**.^[130]^ The procedure allowed both electron‐rich and ‐poor thiophenols to be converted under irradiation with visible light in the presence of caesium carbonate (Scheme 43). Remarkably, the tolerated aryl halides are not limited to activated, electron‐deficient arenes, as thioethers were formed with iodobenzene and toluene; however, a prolonged reaction time was required (20–24 h).

Remarkably fast coupling reactions (1 h) were observed between electron‐deficient aryl halides and electron‐rich thiophenols. In addition, benzylic halides were shown to convert in a similar manner. This procedure allowed the mild and efficient late‐stage functionalization of pharmaceutically active compounds.

**Scheme 44:**
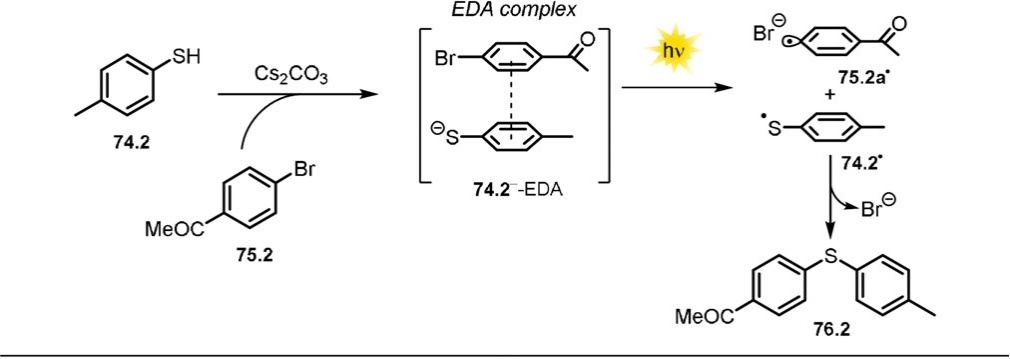
Proposed reaction mechanism for the C−S cross‐coupling reaction of thiophenols and aryl halides via the formation of a visible‐light‐absorbing EDA complex.

The formation of an EDA complex **74.2^−^**‐EDA between thiophenolate **74.2^−^** and aryl halide **75.2** was confirmed by UV/Vis spectroscopy and TD‐DFT calculations. The arising charge‐transfer absorption band allows initiation of the reaction with visible light through an electron transfer from the thiolate anion to the aryl halide, followed by cleavage of the halide anion. The formed thiyl and aryl radical combine to afford the C−S cross‐coupled product **76.2** (Scheme 44).

Based on the perfluoroalkylation of alkenes and alkynes, it was recently shown that the anionic counterpart involved in the formation of the EDA complex can be utilized catalytically.^[131]^ In the presence of base, 2‐bromophenol (**BrPhOH**) was found to promote the visible‐light‐mediated 1,2‐addition of fluoroalkyl iodides to alkenes and alkynes. Noteworthy, although a significant amount of product was formed in the reaction of allylbenzene **77.2** and ethyl difluoroiodoacetate **56.6** in the absence of a phenol catalyst, the yield could be doubled by using a catalytic amount of **BrPhOH**. The use of a more polar solvent gave rise to Heck‐type coupling products **80.1**. Allylphenols, acting themselves as catalysts, could be converted into either the addition product **79.1** or the coupling product **80.1** without adding **BrPhOH**.

**Scheme 45:**
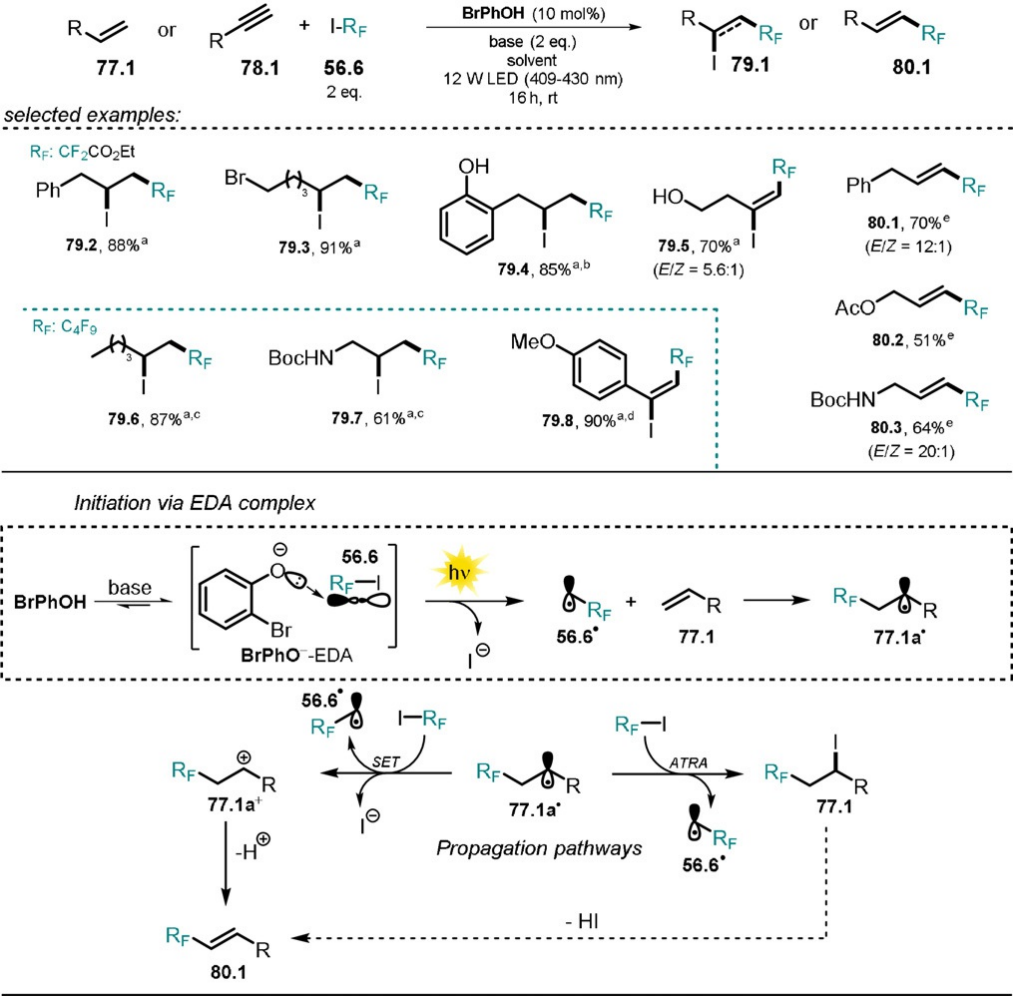
Selected examples of the fluoroalkylation of alkenes and alkynes to afford addition products or Heck‐type coupling products (top). [a] Using KOAc (2 equiv) in DCE. [b] Without **BrPhOH** in dioxane. [c] Cs_2_CO_3_ instead of KOAc. [d] K_2_CO_3_ instead of KOAc. [e] Using K_2_CO_3_ (2 equiv) in DMSO. Proposed reaction mechanism for the visible‐light‐promoted fluoroalkylation using 2‐bromophenol as an initiator (bottom).

Initiation of the reaction is proposed to occur through formation of an EDA complex between phenolate **BrPhO^−^** and the alkylating reagent **56.6**. The photoexcited EDA complex results in the formation of radical **56.6^.^**, which reacts with the olefin **77.1** to yield the radical intermediate **77.1 a^.^**. Depending on the reaction medium, either abstraction of an iodine atom from **56.6** affords the addition product **79.1** or SET with **56.6** gives rise to the cationic intermediate **77.1 a^+^**, which forms the Heck‐type product **80.1** upon deprotonation (Scheme 45).

### 3.7 Organic Anions Promoting the Radical‐Nucleophilic Substitution (S_RN_1) Reaction

**Scheme 46:**
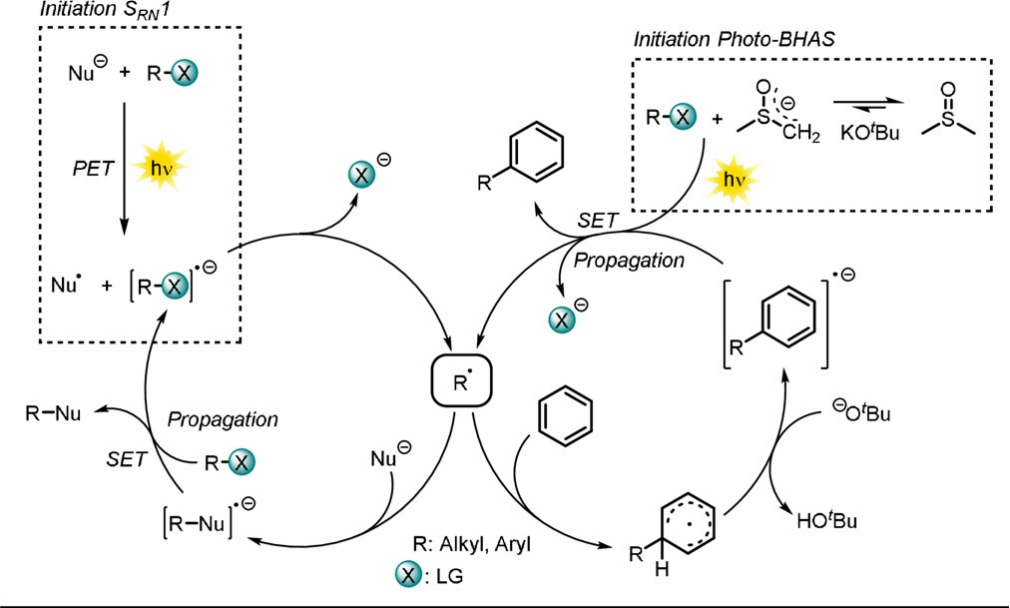
General reaction mechanism for the radical‐nucleophilic substitution (S_RN_1) reaction and for the photoinitiated base‐promoted homolytic aromatic substitution reaction (photo‐BHAS).

In the course of S_RN_1 reactions, radicals and radical anions are formed as intermediates, and chain mechanisms are likely to occur. Proposed for the first time in the 1960s,[^132^, ^133^] the reaction results in nucleophilic substitution of aromatic and aliphatic compounds and tolerates a wide range of nucleophiles and substrates.^[134]^ Initiation is commonly achieved by photoinduced electron transfer from an electron‐rich anionic nucleophile to an electron‐poor acceptor, leading to the open‐shell nucleophile Nu**^.^** and a radical anion [R‐X]**^.−^**. The formation of EDA complexes between the nucleophile and substrate are reported and allow the S_RN_1 reactions to be initiated by using less‐energetic light.^[127]^ Upon mesolytic bond cleavage, the resulting radical R**^.^** is trapped by the nucleophile and forms a radical anion. A single‐electron transfer from the radical anion [R‐Nu]^.−^ to the acceptor R‐X affords the desired substitution product along with another radical anion [R‐X]^.−^, which enables the propagation of a chain reaction (Scheme 46), provided that this SET is thermodynamically favorable.

Closely related to the concept of the light‐induced S_RN_1 reaction is the photoinitiated base‐promoted homolytic aromatic substitution reaction (photo‐BHAS), which affords C−H arylated products starting from aryl or alkyl halides in the presence of a strong base (e.g. KO^*t*^Bu or NaH). The reactive intermediate R^.^ is proposed to add to the arene to form a cyclohexadienyl‐type radical, which is converted into the respective radical anion by deprotonation and eventually gives the arylated product upon SET to R‐X to propagate the chain reaction. In the absence of further additives, it has recently been shown that the dimsyl anion can be excited by visible light and plays a pivotal role in initiating the reaction (see Scheme 46).^[135]^ The initiation of the BHAS reaction was also reported by other photoactivation modes, for example, through PET from an iridium sensitizer to R‐X, or upon excitation with light of an in situ formed photosensitive complex between KO^*t*^Bu and phenanthroline.[^136^, ^137^] Non‐nucleophilic bases are commonly employed to avoid the competing S_RN_1 reaction pathway. Light‐mediated substitutions following the S_RN_1 reaction with organic anions as nucleophiles have been studied extensively and were the subject of recent reviews[^4^, ^134^, ^138^, ^139^, ^140^, ^141^, ^142^] and thus will not be further discussed herein.

### 3.8 Direct Photodecarboxylation of Carboxylates

**Scheme 47:**
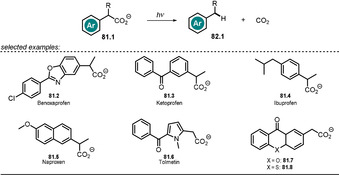
General scheme for the photodecarboxylation of dissociated aryl acetic acids and prominent examples.

In the presence of light, various organic carboxylates are known to undergo photodecarboxylation (PDC) to afford CO_2_ and either a carbanion intermediate (heterolytic cleavage) or an alkyl radical intermediate in combination with a solvated electron (homolytic cleavage). Meiggs et al.^[143]^ performed flash photolysis of sodium phenyl acetate and could prove the formation of a benzyl radical intermediate by transient absorption spectroscopy. The formation of toluene, in addition to polyacids and bibenzyl, may suggest a competing heterolytic bond‐cleavage mechanism. Reaction pathways via high‐energetic carbanion or radical intermediates are favored with compounds bearing stabilizing substituents. Hence, PDC is often observed upon irradiation of dissociated aryl acetic acids **81.1**, thereby generating intermediates that benefit from benzylic stabilization (Scheme 47). The light‐mediated decomposition of carboxylates has been covered in detail in various reviews and thus is beyond the scope of this Review.[^144^, ^145^, ^146^]

Photobases are important initiators of photopolymerization processes. Xanthone and thioxanthone acetic acids (**81.7**, **81.8**) form carbanions upon decarboxylation and have recently received interest as amine‐free alternatives for efficient thiol‐epoxy polymerization.[^147^, ^148^]

### 3.9 Sulfite Anions Used in Photoreactions

**Scheme 48:**
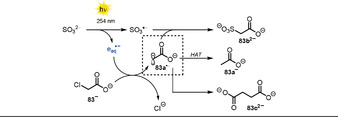
Dechlorination of monochloroacetic acid by solvated electrons produced upon excitation of sulfite anions with UV light.

The ability of cheap and available sulfite salts to generate hydrated electrons upon irradiation renders their use attractive (Scheme 48). The method was successfully applied for the photodegradation of hazardous halogenated pollutants such as monochloroacetic acid^[149]^ **83** and perfluorooctanesulfonate.^[150]^ However, harmful high‐energetic UV light (254 nm) is necessary to photoexcite sulfite anions, and the process efficiency suffers in more complex media because of light attenuation by scattering or competing absorption of other compounds, including the solvent.

## 4 Summary and Outlook

Organic anions and light are a perfect combination to achieve challenging synthetic transformations by acting as either reagents or photocatalysts. Compared to a corresponding neutral molecule, the absorption spectrum of its anion usually exhibits a bathochromic shift and often fluorescence is exclusively observed for the anionic species. This allows photochemical conversions with less‐energetic light, in many cases visible light. Fluorescence quenching studies enable the verification of interactions between substrates and the excited chromophore. The seminal work of Soumillion and co‐workers in this field and their excellent review^[4]^ demonstrated early on the potential of organic anions as strong photoreductants in the dechlorination of arenes and the desulfonylation of sulfonamides using excited 2‐naphtholate. The oxygen‐centered radicals of photoexcited anionic decatungstates allow strong C(sp^3^)−H bonds of non‐prefunctionalized alkanes to be broken to form new carbon bonds. Synergistic approaches of HAT and transition‐metal catalysis have recently found widespread interest and have also enabled asymmetric reactions. In addition to the use of anions as photocatalysts, excited anions have found applications as strong reductants to activate a reaction partner by PET followed by a subsequent conversion of both open‐shell intermediates. Examples are the arylation of azaallylanions or the Heck‐type arylation of vinylphenols. Photoexcited organic anions allow cyclization reactions to yield pyrazoles or participate in ring‐expansion reactions. Moreover, organic anions serve as potent electron‐rich donor molecules for the formation of light‐absorbing EDA complexes.

Overall, the use of photoexcited anions harbors enormous potential for applications in synthetic organic chemistry. We observe increasing research interest in applying photoexcited anions as catalysts or reagents and hope that this Review will stimulate more contributions to this yet underexplored but emerging field, which holds promise for many more exciting applications in organic synthesis.

## Conflict of interest

The authors declare no conflict of interest.

## Biographical Information

*Matthias Schmalzbauer completed his M.Sc. in Chemistry at the University of Regensburg (Germany) in 2016. During his master studies, he joined the group of Prof. König and spent a research period at the UNC (Córdoba, Argentina) under the supervision of Dr. J. I. Bardagí and Prof. R. A. Rossi. He received his Ph.D. from the University of Regensburg in 2020 and is currently a postdoctoral fellow in the König group, with research interests focusing on organic photoredox catalysis*.

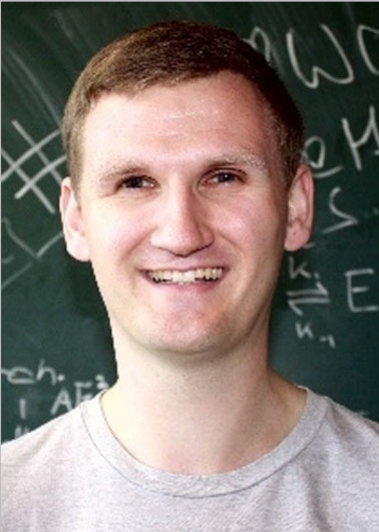

## Biographical Information

*Michela Marcon obtained her master's degree in Chemistry in 2019 at the University of Padova (Italy), under the supervision of Dr. X. Companyó and Dr. L. Dell′Amico. She then joined the group of Prof. König as an Erasmus trainee, working on the photocatalytic generation of carbanions. Currently, she is a PhD student in the König group*.

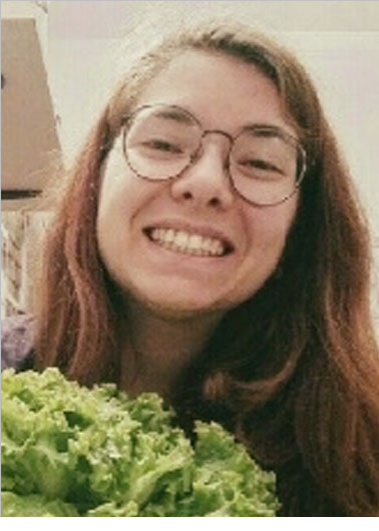

## Biographical Information

*Burkhard König received his Ph.D. from the University of Hamburg and continued his scientific education as a postdoctoral fellow with Prof. M. A. Bennett, Australian National University, Canberra, and Prof. B. M. Trost, Stanford University. Since 1999 he has been a full professor of organic chemistry at the University of Regensburg. His current research interests are synthetic methods utilizing visible light for organic synthesis and applications of photochromic molecules*.

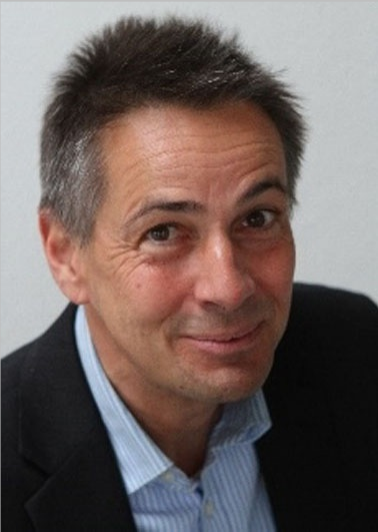

## References

[1] M. A. Fox , Chem. Rev. 1979, 79, 253–273.

[2] L. M. Tolbert , Acc. Chem. Res. 1986, 19, 268–273.

[3] E. Krogh , P. Wan , Top. Curr. Chem. 1990, 156, 93–116.

[4] J.-P. Soumillion , Top. Curr. Chem. 1993, 168, 93–141.

[5] T. O. Baldwin , Structure 1996, 4, 223–228. 8805542 10.1016/s0969-2126(96)00026-3

[6] K. J. Hellingwerf , J. Hendriks , T. Gensch , J. Phys. Chem. A 2003, 107, 1082–1094.

[7] S. Faraji , A. Dreuw , Annu. Rev. Phys. Chem. 2014, 65, 275–292. 24364918 10.1146/annurev-physchem-040513-103626

[8] W. Lee , G. Kodali , R. J. Stanley , S. Matsika , Chem. Eur. J. 2016, 22, 11371–11381. 27362906 10.1002/chem.201600656

[9] A. H. Zimmerman , R. Gygax , J. I. Brauman , J. Am. Chem. Soc. 1978, 100, 5595–5597.

[10] J. P. Soumillion , P. Vandereecken , M. Van Der Auweraer , F. C. De Schryver , A. Schanck , J. Am. Chem. Soc. 1989, 111, 2217–2225.

[11] N. Tamaoki , Y. Takahashi , T. Yamaoka , J. Chem. Soc. Chem. Commun. 1994, 1749–1750.

[12] J. Smid , Angew. Chem. Int. Ed. Engl. 1972, 11, 112–127;

[13] M. J. Kaufman , S. Gronert , D. A. Bors , A. Streitwieser , J. Am. Chem. Soc. 1987, 109, 602–603.

[14] Y. Marcus , G. Hefter , Chem. Rev. 2006, 106, 4585–4621. 17091929 10.1021/cr040087x

[15] H. D. Roth , Top. Curr. Chem. 1990, 156, 1–19.

[16] D. Rehm , A. Weller , Isr. J. Chem. 1970, 8, 259–271.

[17] N. A. Romero , D. A. Nicewicz , Chem. Rev. 2016, 116, 10075–10166. 27285582 10.1021/acs.chemrev.6b00057

[18] B. Legros , P. Vandereecken , J. P. Soumillion , J. Phys. Chem. 1991, 95, 4752–4761.

[19] E. Vander Donckt , J. Nasielski , P. Thiry , J. Chem. Soc. D 1969, 1249–1250.

[20] C. Kerzig , M. Goez , Phys. Chem. Chem. Phys. 2015, 17, 13829–13836. 25929856 10.1039/c5cp01711d

[21] M. Brautzsch , C. Kerzig , M. Goez , Green Chem. 2016, 18, 4761–4771.

[22] M. Schmalzbauer , I. Ghosh , B. König , Faraday Discuss. 2019, 215, 364–378. 30957806 10.1039/c8fd00176f

[23] X. F. Zhang , I. Zhang , L. Liu , Photochem. Photobiol. 2010, 86, 492–498. 20331524 10.1111/j.1751-1097.2010.00706.x

[24] K. Hashimoto , T. Kawai , T. Sakata , Chem. Lett. 1983, 12, 709–712.

[25] E. F. Zwicker , L. I. Grossweiner , J. Phys. Chem. 1963, 67, 549–555.

[26] L. I. Grossweiner , E. F. Zwicker , J. Chem. Phys. 1961, 34, 1411–1417.

[27] K. Kimura , T. Miwa , M. Imamura , Chem. Commun. 1968, 1619–1621.

[28] A. Aguirre-Soto , K. Kaastrup , S. Kim , K. Ugo-Beke , H. D. Sikes , ACS Catal. 2018, 8, 6394–6400.

[29] C. Munkholm , D. R. Parkinson , D. R. Walt , J. Am. Chem. Soc. 1990, 112, 2608–2612.

[30] A. Joshi-Pangu , F. Lévesque , H. G. Roth , S. F. Oliver , L. C. Campeau , D. Nicewicz , D. A. DiRocco , J. Org. Chem. 2016, 81, 7244–7249. 27454776 10.1021/acs.joc.6b01240

[31] E. Speckmeier , T. G. Fischer , K. Zeitler , J. Am. Chem. Soc. 2018, 140, 15353–15365. 30277767 10.1021/jacs.8b08933

[32] F. Speck , D. Rombach , H. A. Wagenknecht , Beilstein J. Org. Chem. 2019, 15, 52–59. 30680038 10.3762/bjoc.15.5 PMC6334793

[33] L. Dell′Amico , A. Vega-Penaloza , J. Mateos , X. Companyo , M. Escudero-Casao , Angew. Chem. Int. Ed. 2020, 10.1002/anie.202006416; 32568437

[34] C. K. Prier , D. A. Rankic , D. W. C. MacMillan , Chem. Rev. 2013, 113, 5322–5363. 23509883 10.1021/cr300503r PMC4028850

[35] C. Streb , Dalton Trans. 2012, 41, 1651–1659. 22183140 10.1039/c1dt11220a

[36] I. Ghosh , J. Khamrai , A. Savateev , N. Shlapakov , M. Antonietti , B. König , Science 2019, 365, 360–366. 31346061 10.1126/science.aaw3254

[37] Y. Y. Loh , K. Nagao , A. J. Hoover , D. Hesk , N. R. Rivera , S. L. Colletti , I. W. Davies , D. W. C. MacMillan , Science 2017, 358, 1182–1187. 29123019 10.1126/science.aap9674 PMC5907472

[38] I. Ghosh , T. Ghosh , J. I. Bardagi , B. König , Science 2014, 346, 725–728. 25378618 10.1126/science.1258232

[39] I. Ghosh , B. König , Angew. Chem. Int. Ed. 2016, 55, 7676–7679; 10.1002/anie.201602349 27198967

[40] J. I. Bardagi , I. Ghosh , M. Schmalzbauer , T. Ghosh , B. König , Eur. J. Org. Chem. 2018, 34–40.

[41] M. Neumeier , D. Sampedro , M. Májek , V. A. de la Peña O'Shea , A. Jacobi von Wangelin , R. Pérez-Ruiz , Chem. Eur. J. 2018, 24, 105–108. 29131437 10.1002/chem.201705326

[42] M. Majek , A. Jacobi Von Wangelin , Acc. Chem. Res. 2016, 49, 2316–2327. 27669097 10.1021/acs.accounts.6b00293

[43] I. Ghosh , L. Marzo , A. Das , R. Shaikh , B. König , Acc. Chem. Res. 2016, 49, 1566–1577. 27482835 10.1021/acs.accounts.6b00229

[44] I. Ghosh , Phys. Sci. Rev. 2019, 4, 20170185.

[45] C. S. Wang , P. H. Dixneuf , J. F. Soulé , Chem. Rev. 2018, 118, 7532–7585. 30011194 10.1021/acs.chemrev.8b00077

[46] J. P. Cole , D.-F. Chen , M. Kudisch , R. M. Pearson , C.-H. Lim , G. M. Miyake , J. Am. Chem. Soc. 2020, 142, 13573–13581. 32662645 10.1021/jacs.0c05899 PMC7849045

[47] J. A. Christensen , B. T. Phelan , S. Chaudhuri , A. Acharya , V. S. Batista , M. R. Wasielewski , J. Am. Chem. Soc. 2018, 140, 5290–5299. 29589754 10.1021/jacs.8b01778

[48] D. Weir , J. C. Scaiano , Chem. Phys. Lett. 1986, 128, 156–159.

[49] A. Samanta , K. Bhattacharyya , P. K. Das , P. V. Kamat , D. Weir , G. L. Hug , J. Phys. Chem. 1989, 93, 3651–3656.

[50] J. C. Scaiano , M. Tanner , D. Weir , J. Am. Chem. Soc. 1985, 107, 4396–4403.

[51] L. J. Johnston , Chem. Rev. 1993, 93, 251–266.

[52] B. R. Arnold , J. C. Scaiano , W. G. McGimpsey , J. Am. Chem. Soc. 1992, 114, 9978–9982.

[53] I. A. MacKenzie , L. Wang , N. P. R. Onuska , O. F. Williams , K. Begam , A. M. Moran , B. D. Dunietz , D. A. Nicewicz , Nature 2020, 580, 76–80. 32238940 10.1038/s41586-020-2131-1 PMC7138348

[54] D. Gosztola , M. P. Niemczyk , W. Svec , A. S. Lukas , M. R. Wasielewski , J. Phys. Chem. A 2000, 104, 6545–6551.

[55] J. Haimerl , I. Ghosh , B. König , J. Vogelsang , J. M. Lupton , Chem. Sci. 2019, 10, 681–687. 30746104 10.1039/c8sc03860k PMC6340401

[56] S. Fukuzumi , K. Ohkubo , T. Suenobu , Acc. Chem. Res. 2014, 47, 1455–1464. 24793793 10.1021/ar400200u

[57] T. Hering , T. Slanina , A. Hancock , U. Wille , B. König , Chem. Commun. 2015, 51, 6568–6571. 10.1039/c5cc01580d 25772087

[58] B. Zilate , C. Fischer , C. Sparr , Chem. Commun. 2020, 56, 1767–1775. 10.1039/c9cc08524f 31998897

[59] K. A. Margrey , D. A. Nicewicz , Acc. Chem. Res. 2016, 49, 1997–2006. 27588818 10.1021/acs.accounts.6b00304

[60] Q. Xu , B. Zheng , X. Zhou , L. Pan , Q. Liu , Y. Li , Org. Lett. 2020, 22, 1692–1697. 31944775 10.1021/acs.orglett.9b04201

[61] P. D. Morse , T. M. Nguyen , C. L. Cruz , D. A. Nicewicz , Tetrahedron 2018, 74, 3266–3272. 30287974 10.1016/j.tet.2018.03.052 PMC6166882

[62] E. Alfonzo , F. S. Alfonso , A. B. Beeler , Org. Lett. 2017, 19, 2989–2992. 28530103 10.1021/acs.orglett.7b01222

[63] K. Wang , L. G. Meng , L. Wang , Org. Lett. 2017, 19, 1958–1961. 28368617 10.1021/acs.orglett.7b00292

[64] K. Tu , T. Xu , L. Zhang , Z. Cheng , X. Zhu , RSC Adv. 2017, 7, 24040–24045.

[65] T. Krappitz , K. Jovic , F. Feist , H. Frisch , V. P. Rigoglioso , J. P. Blinco , A. J. Boydston , C. Barner-Kowollik , J. Am. Chem. Soc. 2019, 141, 16605–16609. 31592659 10.1021/jacs.9b09025

[66] J. Li , Z. Liu , S. Wu , Y. Chen , Org. Lett. 2019, 21, 2077–2080. 30888188 10.1021/acs.orglett.9b00353

[67] P. D. Morse , D. A. Nicewicz , Chem. Sci. 2015, 6, 270–274. 25541590 10.1039/c4sc02331e PMC4273915

[68] H. T. Qin , S. W. Wu , J. L. Liu , F. Liu , Chem. Commun. 2017, 53, 1696–1699. 10.1039/c6cc10035j 28101550

[69] M. Majek , F. Filace , A. J. Von Wangelin , Beilstein J. Org. Chem. 2014, 10, 981–989. 24991248 10.3762/bjoc.10.97 PMC4077370

[70] D. P. Haria , B. König , Chem. Commun. 2014, 50, 6688–6699. 10.1039/c4cc00751d 24699920

[71] V. Srivastava , P. P. Singh , RSC Adv. 2017, 7, 31377–31392.

[72] K. Liang , Q. Liu , L. Shen , X. Li , D. Wei , L. Zheng , C. Xia , Chem. Sci. 2020, 11, 6996–7002.

[73] J. P. Soumillion , P. Vandereecken , F. C. De Schryver , Tetrahedron Lett. 1989, 30, 697–700.

[74] M. Ayadim , J. P. Soumillion , Tetrahedron Lett. 1996, 37, 381–384.

[75] A. H. Dwivedi , U. Pande , J. Photochem. Photobiol. A 2003, 154, 303–309.

[76] J. F. Art , J. P. Kestemont , J. P. Soumillion , Tetrahedron Lett. 1991, 32, 1425–1428.

[77] E. Hasegawa , N. Izumiya , T. Miura , T. Ikoma , H. Iwamoto , S. Y. Takizawa , S. Murata , J. Org. Chem. 2018, 83, 3921–3927. 29537851 10.1021/acs.joc.8b00282

[78] E. Hasegawa , Y. Nagakura , N. Izumiya , K. Matsumoto , T. Tanaka , T. Miura , T. Ikoma , H. Iwamoto , K. Wakamatsu , J. Org. Chem. 2018, 83, 10813–10825. 30015483 10.1021/acs.joc.8b01536

[79] E. Hasegawa , T. Tanaka , N. Izumiya , T. Kiuchi , Y. Ooe , H. Iwamoto , S. Y. Takizawa , S. Murata , J. Org. Chem. 2020, 85, 4344–4353. 32073264 10.1021/acs.joc.0c00038

[80] J. H. Baxendale , Radiat. Res. Suppl. 1964, 4, 114–138.

[81] E. J. Hart , Surv. Prog. Chem. 1969, 5, 129–184.

[82] G. V. Buxton , C. L. Greenstock , W. P. Helman , A. B. Ross , J. Phys. Chem. Ref. Data 1988, 17, 513–886.

[83] D. Zhu , L. Zhang , R. E. Ruther , R. J. Hamers , Nat. Mater. 2013, 12, 836–841. 23812128 10.1038/nmat3696

[84] L. Zhang , D. Zhu , G. M. Nathanson , R. J. Hamers , Angew. Chem. Int. Ed. 2014, 53, 9746–9750; 10.1002/anie.201404328 25044766

[85] I. Ghosh , R. S. Shaikh , B. König , Angew. Chem. Int. Ed. 2017, 56, 8544–8549; 10.1002/anie.201703004 28544442

[86] M. Schmalzbauer , T. D. Svejstrup , F. Fricke , P. Brandt , M. J. Johansson , G. Bergonzini , B. König , Chem 2020, 6, 2658–2672.

[87] S. Mahboobi , S. Dove , A. Sellmer , M. Winkler , E. Eichhorn , H. Pongratz , T. Ciossek , T. Baer , T. Maier , T. Beckers , J. Med. Chem. 2009, 52, 2265–2279. 19301902 10.1021/jm800988r

[88] H. Liu , H. Tang , D. Yang , Q. Ji , Chin. J. Pharm. 2011, 42, 641–644.

[89] M. Raghu , J. Grover , S. S. V. Ramasastry , Chem. Eur. J. 2016, 22, 18316–18321. 27731920 10.1002/chem.201604562

[90] E. Lamy , L. Nadjo , J. M. Saveant , J. Electroanal. Chem. 1977, 78, 403–407.

[91] M. K. Nazeeruddin , R. Humphry-Baker , D. Berner , S. Rivier , L. Zuppiroli , M. Graetzel , J. Am. Chem. Soc. 2003, 125, 8790–8797. 12862473 10.1021/ja021413y

[92] A. Ionescu , E. I. Szerb , Y. J. Yadav , A. M. Talarico , M. Ghedini , N. Godbert , Dalton Trans. 2014, 43, 784–789. 24149586 10.1039/c3dt52077c

[93] S. Y. Takizawa , R. Kano , N. Ikuta , S. Murata , Dalton Trans. 2018, 47, 11041–11046. 30027186 10.1039/c8dt02477d

[94] C. Kerzig , X. Guo , O. S. Wenger , J. Am. Chem. Soc. 2019, 141, 2122–2127. 30672694 10.1021/jacs.8b12223

[95] H. Yin , Y. Jin , J. E. Hertzog , K. C. Mullane , P. J. Carroll , B. C. Manor , J. M. Anna , E. J. Schelter , J. Am. Chem. Soc. 2016, 138, 16266–16273. 27936638 10.1021/jacs.6b05712

[96] Potential is reported versus the Fc^+^/Fc couple and was converted to being versus the SCE by adding +380 mV (see Ref. [97]).

[97] V. V. Pavlishchuk , A. W. Addison , Inorg. Chim. Acta 2000, 298, 97–102.

[98] L. L. Costanzo , S. Pistarà , G. Condorelli , J. Photochem. 1983, 21, 45–51.

[99] Y. Qiao , Q. Yang , E. J. Schelter , Angew. Chem. Int. Ed. 2018, 57, 10999–11003; 10.1002/anie.201804022 29752881

[100] V. De Waele , O. Poizat , M. Fagnoni , A. Bagno , D. Ravelli , ACS Catal. 2016, 6, 7174–7182.

[101] D. Ravelli , M. Fagnoni , T. Fukuyama , T. Nishikawa , I. Ryu , ACS Catal. 2018, 8, 701–713.

[102] K. Suzuki , N. Mizuno , K. Yamaguchi , ACS Catal. 2018, 8, 10809–10825.

[103] C. Tanielian , Coord. Chem. Rev. 1998, 178–180, 1165–1181.

[104] N. Li , J. Liu , B. Dong , Y. Lan , Angew. Chem. Int. Ed. 2020, 59, 20779–20793; 10.1002/anie.202008054 32633859

[105] D. Ravelli , M. Fagnoni , T. Fukuyama , T. Nishikawa , I. Ryu , ACS Catal. 2018, 8, 701–713.

[106] I. B. Perry , T. F. Brewer , P. J. Sarver , D. M. Schultz , D. A. DiRocco , D. W. C. MacMillan , Nature 2018, 560, 70–75. 30068953 10.1038/s41586-018-0366-x PMC6192026

[107] P. J. Sarver , V. Bacauanu , D. M. Schultz , D. A. DiRocco , Y. Hong Lam , E. C. Sherer , D. W. C. MacMillan , Nat. Chem. 2020, 12, 459–467. 32203440 10.1038/s41557-020-0436-1

[108] H. Cao , Y. Kuang , X. Shi , K. L. Wong , B. B. Tan , J. M. C. Kwan , X. Liu , J. Wu , Nat. Commun. 2020, 11, 1–8. 32327665 10.1038/s41467-020-15878-6 PMC7181776

[109] P. Fan , Y. Lan , C. Zhang , C. Wang , J. Am. Chem. Soc. 2020, 142, 2180–2186. 31971787 10.1021/jacs.9b12554

[110] Z. Y. Dai , Z. S. Nong , P. S. Wang , ACS Catal. 2020, 10, 4786–4790.

[111] P. Fan , C. Zhang , L. Zhang , C. Wang , Org. Lett. 2020, 22, 3875–3878. 32356666 10.1021/acs.orglett.0c01121

[112] L. Wang , T. Wang , G. J. Cheng , X. Li , J. J. Wei , B. Guo , C. Zheng , G. Chen , C. Ran , C. Zheng , ACS Catal. 2020, 10, 7543–7551.

[113] Y. Kuang , H. Cao , H. Tang , J. Chew , W. Chen , X. Shi , J. Wu , Chem. Sci. 2020, 11, 8912–8918. 10.1039/d0sc02661a PMC8163369 34123145

[114] K. Liang , T. Li , N. Li , Y. Zhang , L. Shen , Z. Ma , C. Xia , Chem. Sci. 2020, 11, 2130–2135. 10.1039/c9sc06184c PMC8150107 34123301

[115] G. Filippini , M. Nappi , P. Melchiorre , Tetrahedron 2015, 71, 4535–4542.

[116] Q. Wang , M. Poznik , M. Li , P. J. Walsh , J. J. Chruma , Adv. Synth. Catal. 2018, 360, 2854–2868.

[117] M. Li , S. Berritt , L. Matuszewski , G. Deng , A. Pascual-Escudero , G. B. Panetti , M. Poznik , X. Yang , J. J. Chruma , P. J. Walsh , J. Am. Chem. Soc. 2017, 139, 16327–16333. 29019654 10.1021/jacs.7b09394 PMC5737768

[118] T. Zhang , Y. Meng , J. Lu , Y. Yang , G.-Q. Li , C. Zhu , Adv. Synth. Catal. 2018, 360, 3063–3068.

[119] R. S. Suarez , R. G. Segura , Tetrahedron Lett. 1988, 29, 1071–1074.

[120] C. Sánchez-Sánchez , E. Pérez-Inestrosa , R. García-Segura , R. Suau , Tetrahedron 2002, 58, 7267–7274.

[121] Y. R. Luo , Comprehensive Handbook of Chemical Bond Energies, CRC Press, Boca Raton, 2007, p. 71.

[122] K. Maruyama , Y. Kubo , J. Org. Chem. 1985, 50, 1426–1435.

[123] R. Suau , C. Sánchez-Sánchez , R. García-Segura , E. Pérez-Inestrosa , Eur. J. Org. Chem. 2002, 1903–1911.

[124] F. N. Figueroa , A. A. Heredia , A. B. Peñéñory , D. Sampedro , J. E. Argüello , G. Oksdath-Mansilla , J. Org. Chem. 2019, 84, 3871–3880. 30827103 10.1021/acs.joc.8b02984

[125] A. K. Ganguly , S. S. Alluri , D. Caroccia , D. Biswas , C. H. Wang , E. Kang , Y. Zhang , A. T. McPhail , S. S. Carroll , C. Burlein , et al., J. Med. Chem. 2011, 54, 7176–7183. 21916489 10.1021/jm200778q

[126] D. K. Rayabarapu , A. Zhou , K. O. Jeon , T. Samarakoon , A. Rolfe , H. Siddiqui , P. R. Hanson , Tetrahedron 2009, 65, 3180–3188. 20161276 10.1016/j.tet.2008.11.053 PMC2702864

[127] C. G. S. Lima , T. D. M. Lima , M. Duarte , I. D. Jurberg , M. W. Paixão , ACS Catal. 2016, 6, 1389–1407.

[128] G. E. M. Crisenza , D. Mazzarella , P. Melchiorre , J. Am. Chem. Soc. 2020, 142, 5461–5476. 32134647 10.1021/jacs.0c01416 PMC7099579

[129] M. Nappi , G. Bergonzini , P. Melchiorre , Angew. Chem. Int. Ed. 2014, 53, 4921–4925; 10.1002/anie.201402008 24668827

[130] B. Liu , C. H. Lim , G. M. Miyake , J. Am. Chem. Soc. 2017, 139, 13616–13619. 28910097 10.1021/jacs.7b07390 PMC5920654

[131] E. Zhu , X. X. Liu , A. J. Wang , T. Mao , L. Zhao , X. Zhang , C. Y. He , Chem. Commun. 2019, 55, 12259–12262. 10.1039/c9cc06587c 31556412

[132] N. Kornblum , R. E. Michel , R. C. Kerber , J. Am. Chem. Soc. 1966, 88, 5662–5663.

[133] G. A. Russell , W. C. Danen , J. Am. Chem. Soc. 1966, 88, 5663–5665.

[134] R. A. Rossi , A. B. Pierini , A. B. Peñéñory , Chem. Rev. 2003, 103, 71–167. 12517182 10.1021/cr960134o

[135] M. E. Budén , J. I. Bardagí , M. Puiatti , R. A. Rossi , J. Org. Chem. 2017, 82, 8325–8333. 28553981 10.1021/acs.joc.7b00822

[136] Y. Cheng , X. Gu , P. Li , Org. Lett. 2013, 15, 2664–2667. 23688041 10.1021/ol400946k

[137] Z. Xu , L. Gao , L. Wang , M. Gong , W. Wang , R. Yuan , ACS Catal. 2015, 5, 45–50.

[138] R. A. Rossi , J. F. Guastavino , M. E. Budén , in Arene Chemistry: Reactions Mechanisms and Methods for Aromatic Compounds (Ed.: J. Mortier ), Wiley, Hoboken, 2015, pp. 243–268.

[139] J. I. Bardagí , M. E. Budén , R. A. Rossi , Targets Heterocycl. Syst. 2016, 20, 247–282.

[140] M. E. Budén , S. E. Martín , R. A. Rossi , in CRC Handbook of Organic Photochemistry and Photobiology (Eds.: A. Griesbeck , M. Oelgemöller , F. Ghetti ), CRC Press, Boca Raton, 2012, pp. 347–368.

[141] A. B. Peñéñory , J. E. Argüello , in Handb. Synth. Photochem. (Eds.: A. Albini , M. Fagnoni ), Wiley-VCH, Weinheim, 2010, pp. 319–351.

[142] A. Studer , D. P. Curran , Nat. Chem. 2014, 6, 765–773. 25143210 10.1038/nchem.2031

[143] T. O. Meiggs , L. I. Grossweiner , S. I. Miller , J. Am. Chem. Soc. 1972, 94, 7981–7986.

[144] D. Budac , P. Wan , J. Photochem. Photobiol. A 1992, 67, 135–166.

[145] F. Boscá , M. L. Marín , M. A. Miranda , in CRC Handbook of Organic Photochemistry and Photobiology Second *Ed*. (Eds.: W. Horspool , F. Lenci ), CRC Press, Boca Raton, 2003, pp. 64 1–10.

[146] M. Lukeman , in CRC Handbook of Organic Photochemistry and Photobiology Third *Ed*. (Eds.: A. Griesbeck , M. Oelgemöller , F. Ghetti ), CRC Press, Boca Raton, 2012, pp. 715–726.

[147] J. A. Blake , E. Gagnon , M. Lukeman , J. C. Scaiano , Org. Lett. 2006, 8, 1057–1060. 16524267 10.1021/ol052953d

[148] X. Dong , P. Hu , G. Zhu , Z. Li , R. Liu , X. Liu , RSC Adv. 2015, 5, 53342–53348.

[149] X. Li , J. Ma , G. Liu , J. Fang , S. Yue , Y. Guan , L. Chen , X. Liu , Environ. Sci. Technol. 2012, 46, 7342–7349. 22681542 10.1021/es3008535

[150] Y. Gu , W. Dong , C. Luo , T. Liu , Environ. Sci. Technol. 2016, 50, 10554–10561. 27607769 10.1021/acs.est.6b03261

